# Hidden Diversity—A New Speciose Gall Midge Genus (Diptera: Cecidomyiidae) Associated with Succulent Aizoaceae in South Africa

**DOI:** 10.3390/insects13010075

**Published:** 2022-01-10

**Authors:** Netta Dorchin, Stephany van Munster, Cornelia Klak, Rauri C. K. Bowie, Jonathan F. Colville

**Affiliations:** 1 School of Zoology, The George S. Wise Faculty of Life Sciences, Tel Aviv University, Tel Aviv 6997801, Israel; 2 The Steinhardt Museum of Natural History, Tel Aviv University, Tel Aviv 6997801, Israel; 3 Department of Biological Sciences, University of Cape Town, Rondebosch, Cape Town 7700, South Africa; stephvm4@gmail.com; 4 Bolus Herbarium, Department of Biological Sciences, University of Cape Town, Rondebosch, Cape Town 7707, South Africa; cornelia.klak@uct.ac.za; 5 Department of Integrative Biology and Museum of Vertebrate Zoology, University of California, Berkeley, CA 94720, USA; bowie@berkeley.edu; 6 NRF Centre of Excellence, Percy FitzPatrick Institute, University of Cape Town, Rondebosch, Cape Town 7701, South Africa; 7 Statistics in Ecology, Environment and Conservation, Department of Statistical Sciences, University of Cape Town, Rondebosch, Cape Town 7700, South Africa; jonathan.colville@gmail.com; 8 Kirstenbosch Research Centre, South African National Biodiversity Institute, Newlands, Cape Town 7725, South Africa

**Keywords:** Aizoaceae, galls, Lasiopterini, Namaqualand, Ruschioideae, *Ruschiola*, Succulent Karoo

## Abstract

**Simple Summary:**

Succulent Aizoaceae (often called “mesembs” or ice plants) form a dominant component of the Succulent Karoo in southern Africa, constituting one of the most species-rich families within the Greater Cape Floristic Region (GCFR). Despite the diversity and abundance of these plants, the diversity of insects specialized on them has never been surveyed methodically prior to this study. In a three-year study of the galling insects associated with succulent Aizoaceae in South Africa, we found that they support a rich community of gall midges (Diptera: Cecidomyiidae), virtually all of which are new to science. This is not surprising, given that knowledge of the Afrotropical fauna of gall midges is scarce, with most species described in the 1900s. Here, we describe the new genus *Ruschiola* with ten species from succulent Aizoaceae in Namaqualand, the Knersvlakte and the Cedarberg regions of South Africa based on morphological, molecular and life history data. The genetic data were particularly important in this study for differentiating taxa, given that *Ruschiola* species are very similar morphologically. Members of this genus develop in leaf galls or in plant tissues without visible gall formation, and are highly host specific.

**Abstract:**

Aizoaceae (Caryophyllales) constitute one of the major floral components of the unique Greater Cape Floristic Region (GCFR), with more than 1700 species and 70% endemism. Within succulent Aizoaceae, the subfamily Ruschioideae is the most speciose and rapidly diversifying clade, offering potential niches for the diversification of specialized herbivorous insects. Nevertheless, insect diversity on these plants has not been studied to date, and knowledge of gall-inducing insects in the Afrotropics is generally scarce. Our recent observations indicate that succulent Aizoaceae in the GCFR support a rich and largely unstudied community of gall midges (Diptera: Cecidomyiidae). Here, we provide a first report of their diversity with a description of a new genus, *Ruschiola* Dorchin, and ten new species, based on morphological and molecular analyses of material collected during a three-year targeted survey across major GCFR vegetation types. A high degree of morphological uniformity in *Ruschiola* suggests recent diversification and necessitated the use of molecular data and laboratory rearing from host plants to verify species boundaries and host ranges.

## 1. Introduction

Aizoaceae is a large family of mostly leaf-succulent plants with over 1700 described species, the majority of which are confined to the winter rainfall region of southern Africa [1]. The Aizoaceae constitute one of the most species-rich families within the Greater Cape Floristic Region (GCFR) and form a dominant component of the Succulent Karoo, an arid to semi-arid biome mostly found along the west coast of southern Africa that is notable for harboring the world’s richest flora of succulent plants [2]. Approximately 70% of Aizoaceae species are endemic to the Greater Cape Flora, i.e., the xeric Extra Cape Flora [2], which incorporates most of the Succulent Karoo biome [3], as well as the comparatively moister Core Cape Flora [4]. Of the five currently recognized subfamilies of Aizoaceae [5], the Ruschioideae, and particularly the “core ruschioids”, account for most of this diversity, resulting from a remarkably recent and rapid diversification unmatched by any other continental or island plant radiation recorded [6,7]. The Ruschioideae also exhibit a remarkably diverse array of growth forms and climatic and edaphic adaptations within their distribution [8], thereby offering a great number of potential niches for specialized herbivorous insects. Nevertheless, the diversity of insects specialized on these succulent plants has never been surveyed methodically prior to the present study.

Haphazard field observations in South Africa and Namibia by two of us (N.D. and J.F.C.) over the past 20 years revealed that succulent Aizoaceae (subfamilies Ruschioideae and Mesembryanthemoideae) are favorable hosts to a large and essentially unstudied community of gall-inducing insects, in particular gall midges (Diptera: Cecidomyiidae), providing the incentive for our recent systematic study of this group. The Cecidomyiidae constitute one of the largest families of Diptera, with more than 6600 described species in 832 genera [9] and thousands of species that are yet to be discovered and described [9,10,11]. A good example of this is the poorly studied Afrotropical fauna of gall midges that currently constitute less than 200 described species out of thousands that are very likely to be present in this region [12,13]. Many of the named herbivorous cecidomyiids recorded from the Afrotropics were poorly described in the early 1900s based on individuals caught in flight without information on their host associations [13]. More recent taxonomic studies of Afrotropical taxa that conform to modern standards, including details on host associations, are scarce (e.g., [14,15]).

In the Cape Floristic Region (CFR) of South Africa (synonymous with the Core Cape Flora sensu Manning and Goldblatt [4]), standardized sampling of galling insects suggested that sclerophyllous vegetation supports more species of gall inducers than other vegetation types found in neighboring plant biomes [16,17]. However, these studies did not survey the Succulent Karoo biome, or, if they did, were restricted to shrubs and excluded succulents [16]. As such, these studies did not assess the diversity of cecidomyiids on succulent Aizoaceae, for which the Succulent Karoo has been a center of rapid speciation. The Succulent Karoo is also recognized as a center of diversification for several other insect groups [18], such as bees [19], Mantophasmatodea [20] and Hopliini beetles [21].

Because many of the gall midges on succulent Aizoaceae develop in plant tissues without obvious gall formation, their presence cannot be easily recognized in the field through simple screening of the vegetation, but rather necessitates targeted sampling and direct rearing from the plants in the laboratory. Our multiple-year survey of succulent Aizoaceae from several different vegetation types within southern Africa, including Fynbos, Renosterveld, Nama Karoo and Succulent Karoo, clearly demonstrated that the greatest diversity and abundance of galling insects on these plants are found in the Succulent Karoo and that the overwhelming majority are gall midges. These observations are in accordance with the notions that xeric habitats have been the centers of adaptive radiation for many gall midges and that the distribution patterns of galling insects in general are driven primarily by the abundance of gall midges [22,23]. Our findings also support the hypothesis that plant diversity, and particularly the presence of species-rich plant families, provides the opportunity for the diversification of gall inducers [17,22,24,25].

Here, we describe the new cecidomyiid genus *Ruschiola* Dorchin and ten new species within it from succulent Aizoaceae in South Africa. The newly described genus belongs to the tribe Lasiopterini, the greatest diversity of which occurs in deserts and saline habitats of the eastern hemisphere, primarily on plants of the family Chenopodiaceae [9,26]. The new genus is morphologically rather uniform, possibly due to relatively recent and rapid radiation, thus requiring genetic data to confirm species boundaries and host associations. It appears to be restricted to the subfamilies Mesembryanthemoideae and Ruschioideae of the Aizoaceae (commonly known as “mesembs” or ice plants), with most of the diversity associated with the core ruschioids (tribes Ruschieae and Drosanthemeae). All known species in this genus develop in the leaves of their host plants, with or without obvious or visible gall formation.

## 2. Materials and Methods

### 2.1. Insect Collecting, Rearing and Preservation

**Figure 1:**
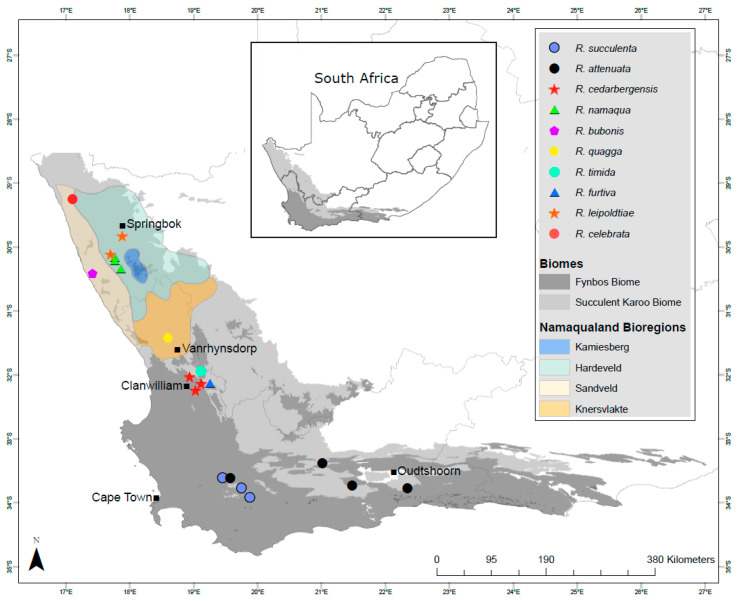
Sampling sites of the ten described *Ruschiola* species in the Fynbos and Succulent Karoo biomes of the Greater Cape Floristic Region of South Africa. Namaqualand bioregions are from Cowling et al. [8] (the northern bioregions of the Richtersveld and southern Namib Desert are not shown).

The field collection of galls and other plant material was conducted over three years, from 2017 to 2019, at multiple sites in the Western and Northern Cape provinces of South Africa, including the three major biodiversity hotspots for succulent Aizoaceae, i.e., the Richtersveld, Knersvlakte and Little Karoo, as well as the major vegetation types within the GCFR [2,4]. Collecting was performed mainly during the austral winter and spring (July–September), which constitutes the peak growth season for succulent Aizoaceae in South Africa, and several sampling excursions were also conducted at other times of the year (January–April). The sampling included multiple field trips to sites in Namaqualand, the Knersvlakte, West Coast, Overberg and Little Karoo (Figure 1).

Due to severe drought conditions that occurred during most of the study period, only the southernmost part of the Richtersveld (Vyftienmyl se Berg Inselberg in southern Richtersveld) was visited. More than 60 sites were visited repeatedly to gain insight into the abundance and phenology of the gall midges and to sample putative host plants across the major clades of succulent Aizoaceae [27,28]. Vouchers of host plants were prepared in a plant press to aid identification, oven dried as necessary, and are deposited in the Bolus Herbarium (BOL), Department of Biological Sciences, University of Cape Town.

Galls and other plant material were collected in mesh bags in the field and placed in ventilated rearing cages on the same day to enable the rearing of adult gall midges. A subset of each gall type was dissected under a stereo microscope in the laboratory to document the developmental stage of the galls and to obtain the larvae and pupae of the gall midges for morphological study. Adults, larvae and pupal exuviae were preserved in 70% ethanol and mounted on permanent microscope slides in Euparal according to the method detailed in Gagné [29]. Pupae were preserved in 70% ethanol and then prepared for scanning electron microscope imaging via chemical drying and gold sputtering. A few adults of some species were also double mounted on micro pins to preserve their color pattern, created by the thick cover of black and white scales on their body. Samples of each morpho-species (mostly adults, occasionally pupae or larvae) were preserved in 99% ethanol for genetic analysis.

### 2.2. Taxonomy

Morphological structures of the gall midges were studied and illustrated with the aid of a drawing tube mounted on a Leica DM1000 compound microscope (Leica Microsystems, Wetzlar, Germany). Some structures were photographed with a Leica DFC495 camera mounted on a Leica M205 C stereo microscope (Leica Microsystems, Wetzlar, Germany). Pupae were studied on a ThermoScientific Phenom XL scanning electron microscope (Thermo Fisher Scientific Inc., Waltham, MA, USA). Terminology for adult morphology follows McAlpine [30]; terminology for wing venation follows Cumming and Wood [31], and terminology for immature morphology follows Gagné [32]. Specific terminology for the lasiopterine ovipositor follows Dorchin [33].

Holotypes are deposited in the Iziko South African Museum, Cape Town, South Africa (SAMC). Paratypes are deposited in SAMC and in the Steinhardt Museum of Natural History, Tel Aviv University, Israel (SMNHTAU), except for representative paratypes, which are deposited at the Zoologisches Forschungsinstitut und Museum Alexander Koenig, Bonn, Germany (ZFMK), and the Essig Museum of Entomology, Berkeley, CA, USA (EMEUC), as detailed in the species descriptions below. All specimens are mounted on permanent microscope slides in Euparal, except for pupae, which are mounted on SEM stubs. All localities mentioned in the examined sections of the species descriptions are in South Africa.

### 2.3. Molecular Methods

The dataset analyzed included 47 individuals of 10 putative species within the newly described genus and seven outgroup taxa from other Lasiopterini genera. Our aim with this analysis was to test the validity of the new genus and species and establish the host ranges of species that appeared to have multiple hosts. GenBank accession numbers for the samples included here are provided in Table 1.

Genomic DNA was extracted from whole individual adults or immature midges using the Genaid Genomic DNA Mini Kit (Genaid, Taipei, Taiwan) or the Qiagen DNeasy Kit (Qiagen, Valencia, CA, USA). A fragment of approximately 680 base pairs of the mitochondrial COI gene was PCR amplified with the primers LCO1490 and HCO2198 [34]. PCR conditions consisted of 10 min denaturation at 95 °C followed by 35 cycles of: 30 s denaturation at 95 °C, 1 min annealing at 50 °C, 1 min extension at 72 °C and final extension at 72 °C for 4 min. PCR reactions were performed in a 2720 Thermal Cycler (Applied Biosystems, Foster City, CA, USA) and products were purified using an EXO-SAP enzymatic cleanup (Thermo Scientific, Vilnius, Lithuania). Sequencing in both directions was performed on an ABI PRISM 3730xl DNA analyzer at Hy Laboratories, Rehovot, Israel. Forward and reverse strands were combined using Sequencer 4.7 (Gene Codes) and all sequences were translated into amino acids to check that no stop codons were present.

### 2.4. Phylogenetic Analysis

The loci were aligned using MAFFT [35] and a maximum likelihood phylogeny constructed in RAXML v8.2.12 [36] on the CIPRES portal [37]. Seven outgroups from other Lasiopterini genera were selected based on Dorchin et al. [38] and the phylogeny was rooted on *Asteromyia carbonifera*, belonging to the sister tribe, Alycaulini. Node support was evaluated using the rapid bootstrapping procedure implemented in RAXML. A GTR model of nucleotide substitution was applied to each codon position independently. Uncorrected pairwise sequence divergences were calculated using Paup* [39].

## 3. Results

### 3.1. Molecular Results

**Figure 2:**
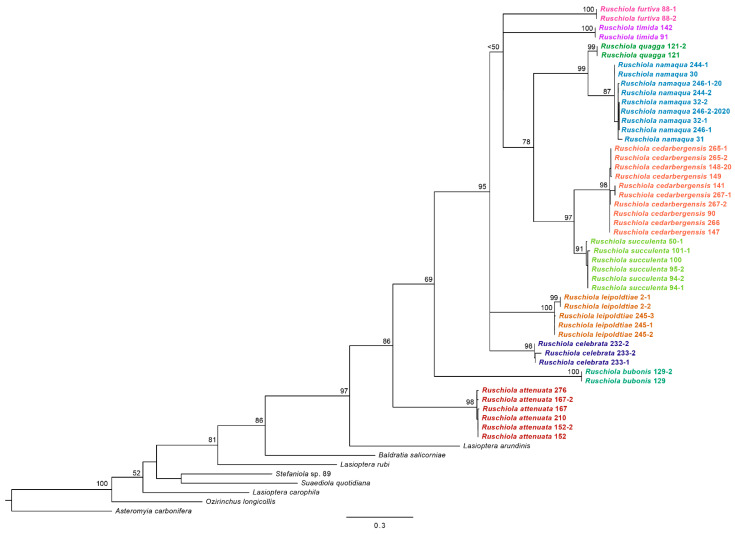
RAXML phylogenetic tree for the ten described *Ruschiola* species, color coded based on sequences of a subsection of the mitochondrial COI gene. Bootstrap support values are shown next to the nodes.

Our molecular analysis verified the monophyly of *Ruschiola* and of all ten species described here (bootstrap values of 87–100%) (Figure 2). Mean intraspecific sequence divergence was 0.0–1.1%, and mean interspecific divergence among *Ruschiola* species was 2.6–10.2% (Table 2). Sequence divergence between *Ruschiola* and the Lasiopterini outgroups used here averaged at 10.4%. *Ruschiola attenuata* from *Mesembryanthemum splendens* was retrieved as sister to all other species included here that develop in Ruschioideae host plants, paralleling the sister position of Mesembryanthemoideae relative to the Ruschioideae within Aizoaceae [5,27]. *Ruschiola attenuata* is also the only species that is relatively easily to distinguish from other *Ruschiola* species based on adult morphology. The high degree of morphological uniformity among other *Ruschiola* species makes it difficult to distinguish between them without genetic data and detailed information on host–plant associations, which may be indicative of recent diversification.

### 3.2. Ruschiola Dorchin, New Genus

Type species: *Ruschiola succulenta* Dorchin and van Munster

**Figure 3:**
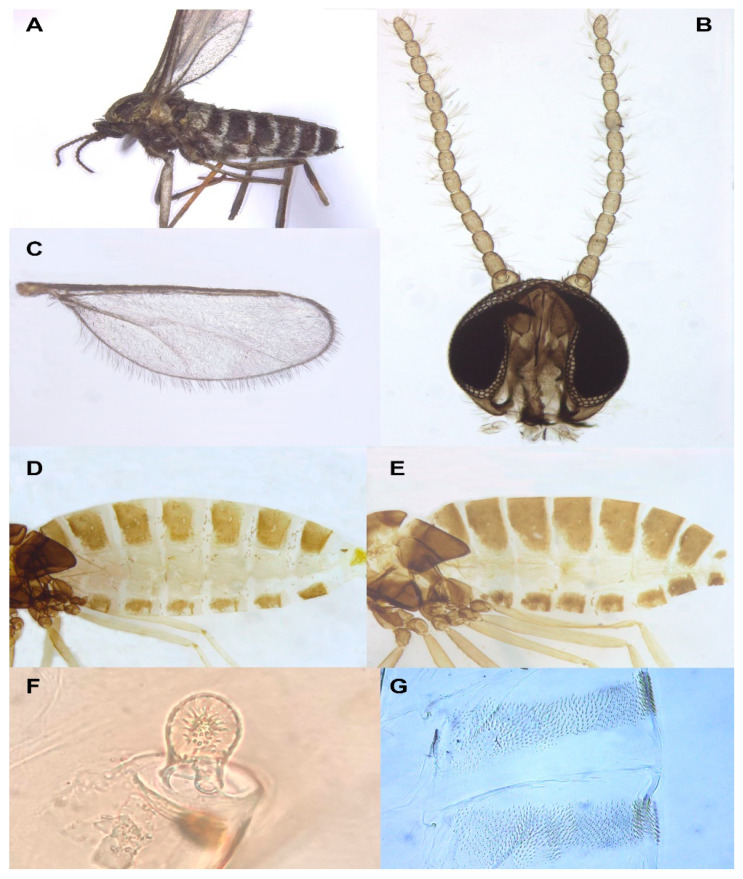
*Ruschiola* spp. (**A**) *R. namaqua*, habitus. (**B**) *R. succulenta*, head. (**C**) *R. namaqua*, wing. (**D**) *R. succulenta*, female abdomen. (**E**) *R. succulenta*, male abdomen. (**F**) *R. succulenta*, pupal prothoracic spiracle, encasing looped trachea. (**G**) *R. succulenta*, pupal abdominal segments, inset.

**Figure 4:**
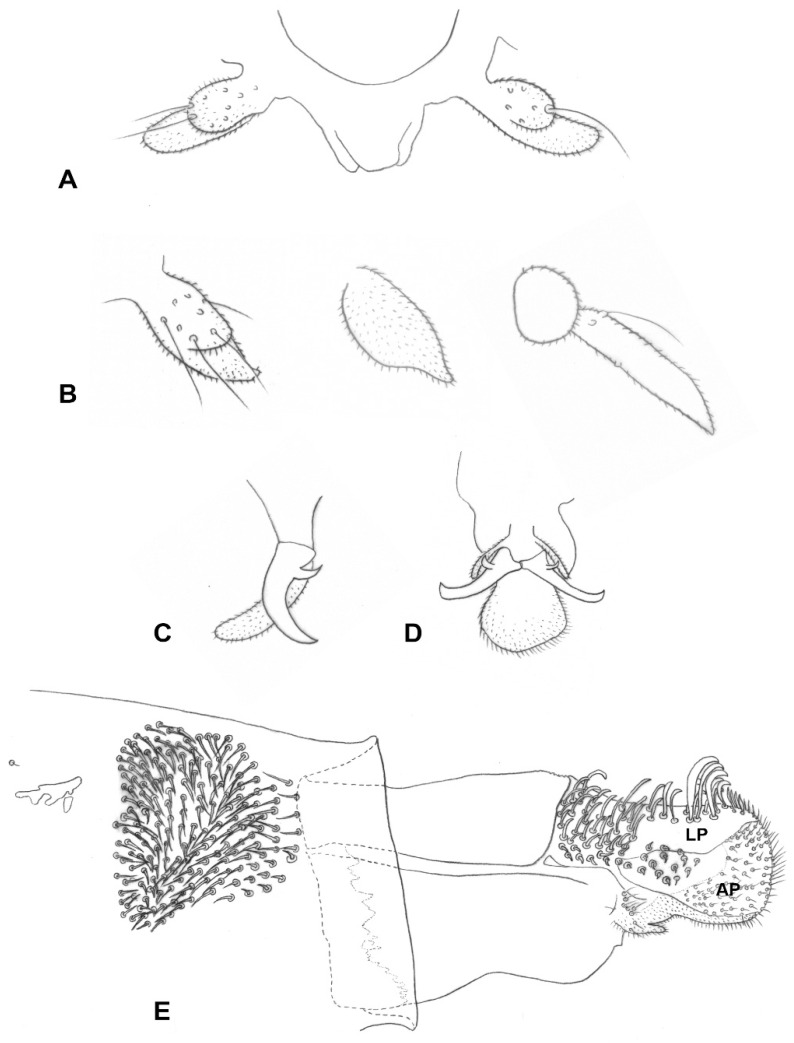
*Ruschiola succulenta*. (**A**) Palpi. (**B**) Variation in palpus morphology. (**C**) Acropod, lateral. (**D**) Acropod, ventral. (**E**) Ovipositor, lateral. LP: lateral plate; AP: apical lamella.

**Diagnosis**: *Ruschiola* is a striking, morphologically uniform genus of medium to large-sized lasiopterines. The adult body is densely covered by black and white scales, whereby abdominal tergites are almost completely black, with only a thin transverse line of white scales along the posterior margin (Figure 3A). This contrasts with the typical color pattern of most lasiopterine genera from Chenopodiaceae (e.g., *Baldratia* Kieffer, *Stefaniola* Kieffer, *Careopalpis* Marikovskij), in which scales on the abdominal tergites form two or three black triangles on a white background (or occasionally vice versa). The number of antennal flagellomeres is irregular within a species, as is also usual for *Lasioptera* Meigen, *Baldratia* and *Stefaniola*, and contrary to the invariable number of 10 flagellomeres in *Careopalpis* and *Suaediola* Dorchin. Palpi are one or two segmented and their shape may vary considerably within the same species and individual (e.g., Figure 4B). Vein R_4+5_ of the wing is notably longer than in most other lasiopterine genera, joining C at about three quarters of the wing length rather than around its mid-length (Figure 3C). The ovipositor is much shorter than that of *Lasioptera* and *Ozirhincus* Rondani, but longer than that of most lasiopterine genera from Chenopodiaceae. It is very uniform within the genus, with a well-developed, heavily sclerotized lateral plate that does not form a clear aculeus posteriorly, contrary to the distinct, ventrally curved aculeus in *Baldratia* and *Careopalpis*, straight aculeus in *Stefaniella* Kieffer, or dorsally curved aculeus in *Stefaniola*, *Izeniola* Fedotova and *Suaediola*. Pupae have well-developed antennal horns but no facial horns, and larvae have a bidentate sternal spatula, conspicuous and long shafted in some species to vestigial and barely visible in others.

The genus currently includes the ten species described here, but many more are likely to be found as the full diversity of Aizoaceae host plants in South Africa and Namibia is yet to be explored. All known *Ruschiola* species develop in conspicuous leaf galls or in leaf tissues without visible external deformation. Many plants that host *Ruschiola* species are also inhabited by *Asphondylia* Loew species, which gall buds or flowers rather than leaves, and will be dealt with elsewhere. The evolutionary relationships between *Ruschiola* and other genera in the Lasiopterini are unclear at present and warrant further study.

Adult. Head (Figure 3B): Eye facets round; gap between eyes on vertex 0–2 facets wide. Antenna: scape cylindrical, pedicel globose, both densely covered by long, dark scales; flagellomeres 10–18, barrel shaped to nearly quadrate (Figure 3B), 1–2 times as long as wide, apical flagellomere either shorter or longer than preceding flagellomeres, rounded or tapered apically, often consisting of two merged flagellomeres; each flagellomere with two whorls of appressed circumfilla with 1–2 longitudinal connections and two more-or-less ordered whorls of strong setae; setae of distal whorl strongly curved at base, stronger, longer and originating from larger sockets than setae of proximal whorl; flagellomeres evenly setulose elsewhere. Frontoclypeal membrane with many long hair-like setae and scales. Palpi 1–2 segmented, shape may vary within species and individual. Labella diminutive to well developed.

*Thorax*: Wing (Figure 3C) densely covered by delicate setulae, posterior margin fringed by long, delicate hair-like setae; C broken beyond junction with R_4+5_; R_4+5_ reaching C at about three quarters of wing length; C and R_4+5_ thick, densely covered by black scales to meeting point; M present as fold; CuA_1_ and CuA_2_ form a fork. Legs: Tarsal claws toothed on all legs, tooth curved close to base; empodia as long as or much longer than bend in claws; pulvilli about half as long as claws.

*Female abdomen* (Figure 3D): Densely covered by scales; tergites mostly black, except for thin stripe of white scales along posterior margin. Tergites 1–7 rectangular, with anterior pair of sensory setae, posterior row of strong setae, and evenly distributed scales; tergite 8 with considerably reduced pigmentation or not differentiated from surrounding membrane, without vestiture other than anterior pair of sensory setae. Sternites 2–6 rectangular, without anterior sensory setae, with few setae forming posterior row along pigmented section and several setae medially; sternite 7 same but setose throughout; sternite 8 with small patch of pigmented area or undifferentiated from surrounding membrane. Ovipositor (Figure 4E): Segment 8 with large lateral group of straight to slightly curved setae on prominent sockets. Segment 9 with pigmented rod-like sclerite along segment. Cercal segment in straight angle relative to segment 9, with ventral field of setulae, strongly sclerotized lateral plate harboring dozens of curved setae on prominent sockets to mid-length, group of shorter setae extending ventrally to unpigmented area, and eight to ten conspicuously long, curl-like setae on distal half, followed by field of shorter, upright acicular setae extending to apical lamella; apical lamella short-cylindrical to rectangular, barely longer than lateral plate, setose and setulose.

**Figure 5:**
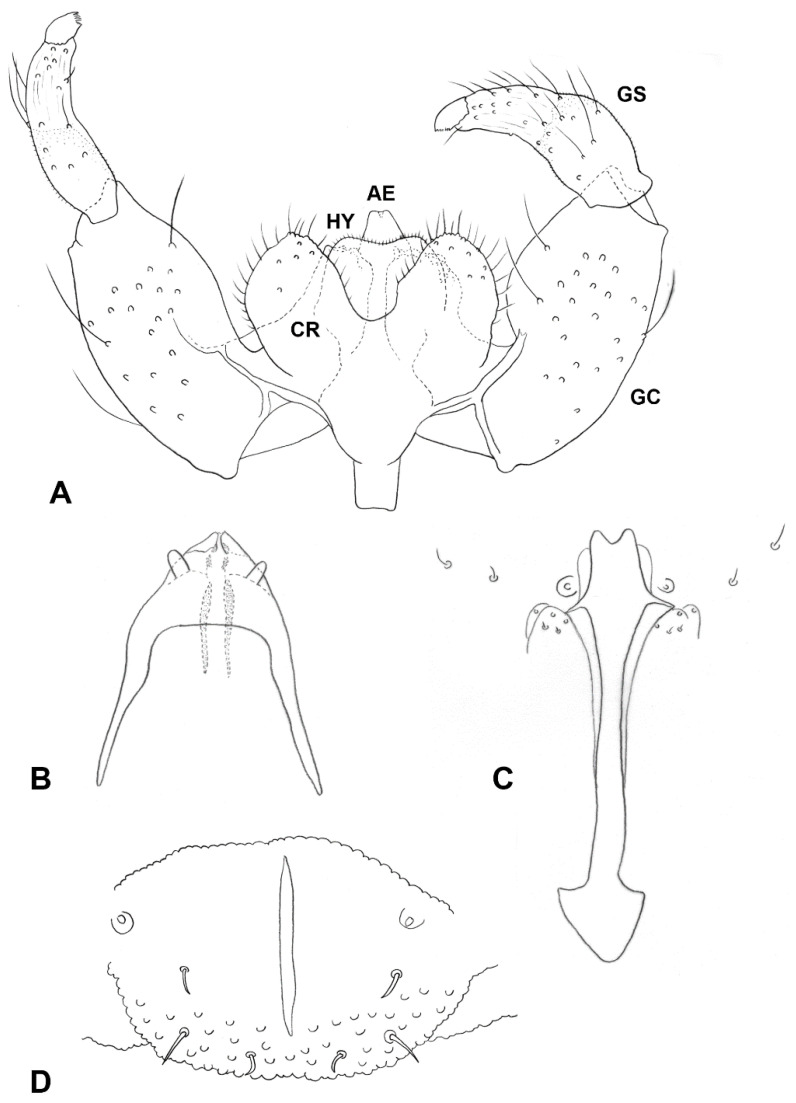
*Ruschiola succulenta*. (**A**) Male terminalia, dorsal. (**B**) Larva, head. (**C**) Larva, spatula and associated papillae. (**D**) Larva, terminal abdominal segment. AE: aedeagus; CR: Cerci; GC: gonocoxite; GS: gonostylus; HY: hypoproct.

*Male abdomen* (Figure 3E): Tergites 1–7 rectangular, vestiture as in female; pigmented part of tergite 8 greatly reduced or tergite completely undifferentiated from surrounding tissue. Sternites 2–7 as in female, pigmented area of sternite 8 considerably smaller than preceding; all sternites nearly evenly setose, denser setation on posterior ones. *Terminalia* (Figure 5A): Gonocoxite widest at mid-length; mediobasal lobe wide, robust, almost same width throughout length, with several short lobes apically, densely setose. Gonostylus widest at proximal third, with wide or medium-sized apical tooth; evenly or partially setulose, grooved elsewhere. Hypoproct rounded, truncated, or with shallow depression apically; shape varies within same species, setulose. Cerci separated almost to base, setose and setulose.

**Larva** (third instar): Light yellow to bright orange. Integument covered by verrucae. Spatula bidentate, conspicuous, with well-developed shaft in some species (e.g., Figure 5C) to vestigial and barely visible in others. Sternal papillae usually asetose; four lateral papillae, asetose or two of which with very short setae, pleural papillae with distinct setae; three terminal papillae on each side, with long setae (Figure 5D).

**Pupa**: Light to dark orange. Antennal bases well developed into tapered horns, parallel or splayed; no facial horns or papillae; short cephalic setae on elevated bases; prothoracic spiracle short and wide, with trachea forming loop inside spiracle (Figure 3F). Basal part of facial area on each side with conspicuous lobe extending laterally. Dorsum of abdominal segments with wide transverse fields of tapered spicules across mid-section (Figure 3G).

**Etymology**: The genus name is feminine, combining *Ruschia* Schwantes, a plant genus of 213 leaf succulent, perennial, shrubby species [1], which hosts the greatest diversity of gall midge species among the Aizoaceae, with the diminutive suffix ‘ola’.

### 3.3. Ruschiola succulenta Dorchin and van Munster, New Species

**Host plants**: *Ruschia caroli* (L. Bolus) Schwantes, *R. pungens* (A. Berger) H. Jacobsen, *Lampranthus haworthii* (Donn ex Haw.) N.E.Br.

**Figure 6:**
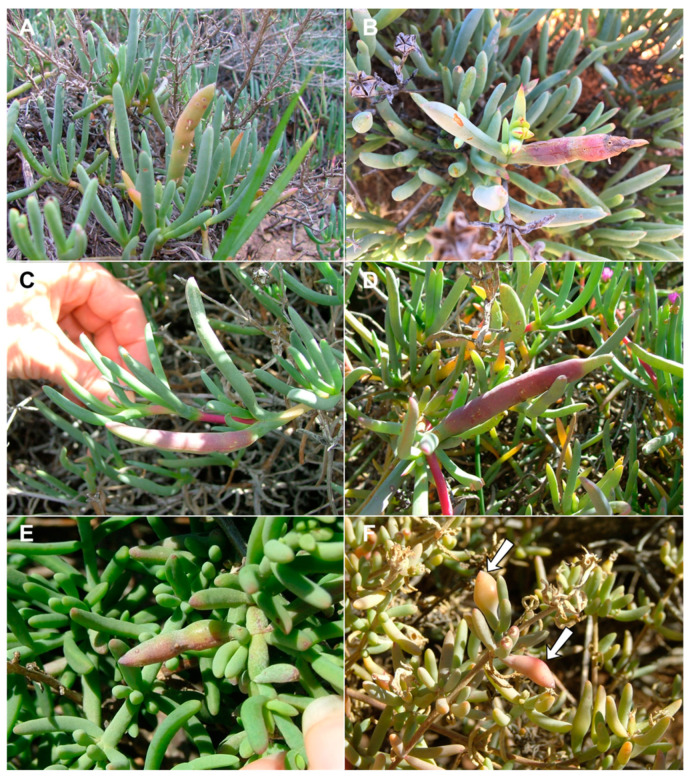
*Ruschiola* galls. (**A**,**B**). *R. succulenta* on *Ruschia caroli*. (**C**,**D**). *R. succulenta* on *Lampranthus haworthii*. (**E**,**F**). *R. attenuata* on *Mesembryanthemum splendens*. Arrows denote galled leaves.

**Gall and biology**: This species develops in swollen, succulent leaf galls, usually 2–6 cm long and 1–1.5 cm wide (Figure 6A–D). The galls may be green but are usually reddish, occasionally with longitudinal cracks on most of the leaf area. Each gall may contain 20–30 larvae in vaguely defined, tunnel-like chambers along the longitudinal axis of the leaf, the walls of which are drier than the surrounding succulent tissues encompassing the rigid middle part of the gall. Galls on *Lampranthus haworthii* are often smaller and less conspicuous than those on *Ruschia caroli* and *R. pungens*, and the larval chambers in them are embedded in rigid, black tissue (probably fungal mycelia). The galls are heavily parasitized by several parasitoid Hymenoptera, including polyembryonic Platygastridae. The species is very common, and its galls can be found in large numbers at some sites, especially on *R. caroli*. Adults emerged mainly in spring (August–September), but galls containing mature larvae and pupae were also found in April, suggesting that the species completes at least two generations a year during the fall and winter months. Full-sized galls were also observed on *R. caroli* in summer (January), but they contained only first-instar larvae. This suggests that the larvae that hatch from eggs in spring (September–October) spend several months in the plant tissues before the galls become apparent and take several additional months to mature and pupate. The galls on all three host plants yielded a second, much smaller *Lasioptera*-like species, whose smaller and more delicate larvae and pupae were found alongside the robust larvae and pupae of *R. succulenta*. This second lasiopterine appears to be an inquiline and will be treated in a future publication, together with congeners we reared from several other succulent Aizoaceae.

**Adult description**: Head, thorax and lateral parts of abdomen white; legs black; wing surface covered by dark hair-like setae, with thick black setae on C up to meeting point with R_4+5_. Abdominal tergites almost entirely black, except for thin transverse line of white scales along posterior margin. Abdominal pleura white, each with small black spot.

*Head* (Figure 3B): Gap between eyes on vertex one to two facets wide. Antennal flagellomeres 11–15 in female (*n* = 30), 10–12 in male (*n* = 16), number often differs between antennae of same individual; cylindrical to barrel shaped, progressively shorter; two adjacent flagellomeres often fused; apical flagellomere slightly pointed or evenly rounded apically. Palpus two segmented; segment 1 globose or slightly longer than wide, setulose and bearing several long setae and scales; segment 2 usually subtending and partially fused with segment 1 (Figure 4A), cylindrical, slightly tapered, setulose; occasionally vestigial or considerably longer than segment 1 (Figure 4B).

*Thorax*: Wing as in generic diagnosis; length 1.78–2.80 mm in female (*n* = 40), 1.37–2.36 mm in male (*n* = 17). Legs densely covered by black scales; claws evenly curved, with strongly curved tooth; empodia considerably longer than bend in claws (Figure 4C,D); pulvilli reaching about mid-length of claws (Figure 4D).

*Female abdomen* (Figure 3D): Tergites 1–7 with anterior pair of minute sensory setae and posterior row of long setae; tergite 8 hardly differentiated from surrounding membrane, with small, irregular patch of pigmented area, or completely unpigmented, pair of sensory setae anterior to pigmented area the only vestiture (Figure 4E). Sternites without proximal sensory setae; sternites 2–6 with unpigmented patch medially, group of long setae medially and posterior row of long setae; sternite 7 evenly pigmented, with long setae on distal two thirds; sternite 8 completely undifferentiated from surrounding membrane. Ovipositor (Figure 4E): Segment 9 with pigmented patches proximally and strongly pigmented rod-like sclerite along segment. Cercal segment with heavily sclerotized lateral plate, slightly tapered apically but not differentiated into clear aculeus; lateral plate bearing about 30 long, curved setae up to mid-length, with additional group of 10–20, shorter setae extending ventrally to unpigmented area, and eight to ten conspicuously long, thick, erect, curl-like setae on distal half, followed by group of shorter, upright acicular setae to tip of lateral plate, extending ventrally to apical lamella; area of lateral plate ventral to curl-like setae bare, sheathing almost half height of apical lamella before abrupt attenuation toward tip. Apical lamella rectangular, barely longer than lateral plate, strongly setose and setulose along distal half. Base of cercal segment with group of strong setae proximal to setulose hypoproct, and densely setulose ventral area extending to tip of apical lamella.

*Male abdomen* (Figure 3E): Tergites 1–7 as in female; tergite 8 much smaller, without posterior row of setae. Sternites 2–7 as in female, pigmented area of sternite 8 smaller than preceding. *Terminalia* (Figure 5A): Gonocoxite widest at mid-length, with numerous strong setae more or less evenly distributed; mediobasal lobe much shorter than aedeagus, almost same width throughout length or slightly narrows to apex in dorsal view, apex rugose, with multiple short setae on bulging bumps. Gonostylus widest at proximal third, setulose on proximal half and furrowed beyond both dorsally and ventrally, with wide apical tooth and numerous long, evenly distributed setae. Aedeagus wide, truncated apically in dorsal view, triangular and pointed anteriorly in lateral view. Hypoproct entire, truncated, or with very shallow apical depression, two setae apically and setulose throughout. Cerci robust, strongly setose and setulose.

**Larva** (third instar): Antennae about twice as long as wide. Posterolateral apodemes at least twice as long as head capsule (Figure 5B). Spatula (Figure 5C) well developed, with short, apically rounded teeth separated by round notch; long, narrow shaft, widens posteriorly. On each side of spatula asetose sternal papilla, four lateral papillae, two of which with minute setae, and one asetose ventral papilla; pleural and dorsal papillae with long setae. Terminal abdominal segment (Figure 5D) on each side with three papillae bearing long setae; anus ventral.

**Figure 7:**
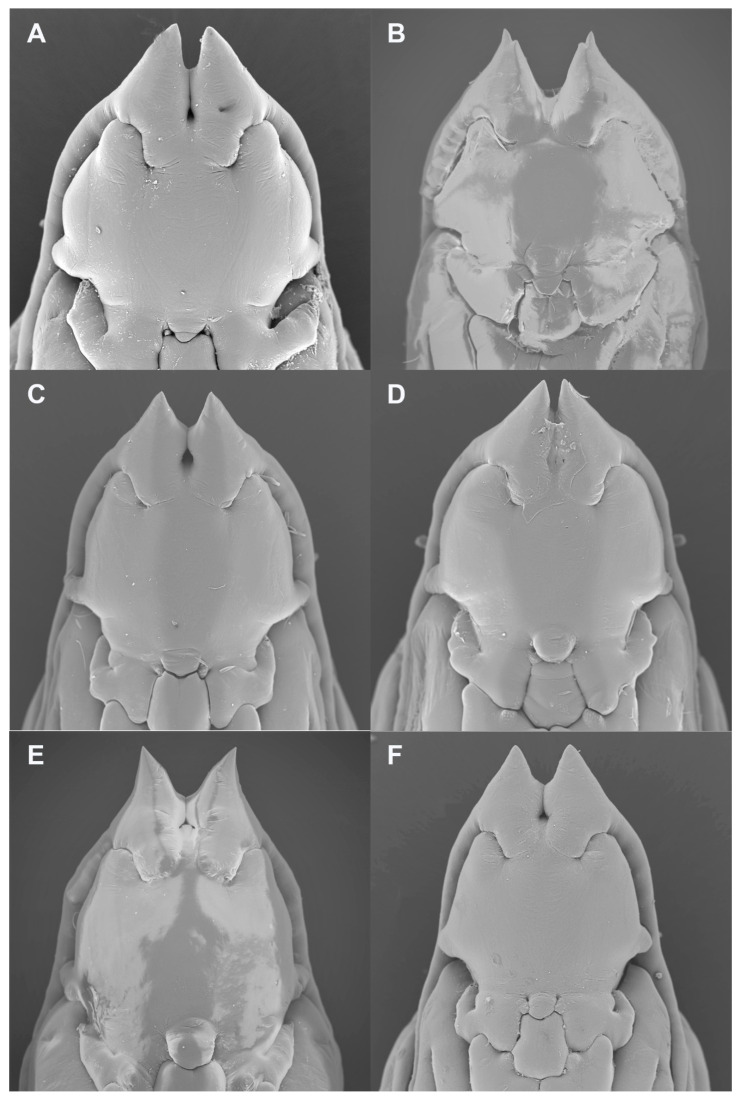
*Ruschiola* pupal heads, frontal. (**A**) *R. succulenta*. (**B**) *R. attenuata*. (**C**) *R. cedarbergensis*. (**D**) *R. namaqua*. (**E**) *R. bubonis*. (**F**) *R. leipoldtiae*.

**Pupa** (Figure 7A). Antennal horns straight, parallel along medial margins.

**Distribution:** Common in the winter rainfall areas of the Western Cape, mainly on *R. caroli*; collected in Worcester, Eilandia near Robertson, and the Vrolijkheid Nature Reserve.

**Etymology:** The species epithet is a feminine adjective, referring to the typical succulent, sausage-like galls induced by this species.

**Type material:** HOLOTYPE: ♀, South Africa, Western Cape, Karoo Desert National Botanical Garden, Worcester (33°36′33″ S, 19°27′01″ E), 20.ix.17, N. Dorchin, S. van Munster and C. Klak, ex leaf gall on *Ruschia caroli*. On permanent microscope slide in Euparal. Deposited in SAMC. PARATYPES: 6♀, 2♂, same data as holotype (1♀ ZFMK); 5♀, 4♂, Karoo Desert National Botanical Garden, Worcester, 25.viii.17, N. Dorchin, S. van Munster and C. Klak, ex *Ruschia caroli* (1♀ EMEUC); 2♀, 3♂, Karoo Desert National Botanical Garden, Worcester, 20.ix.17, N. Dorchin and S. van Munster, ex *Lampranthus haworthii*; 5♀, 1♂, Eilandia, Robertson, 15 km W, Rt60 (33°46′15″ S, 19°44′53″ E), 20.ix.17, N. Dorchin and S. van Munster, ex *Ruschia pungens*; 5♀, 4♂, Eilandia, Robertson, 15 km W, Rt60, 4.ix.18, N. Dorchin and S. van Munster, ex *Ruschia caroli*; 5♀, 4♂, Eilandia, Robertson, 15 km W, Rt60, 6.ix.2018, N. Dorchin and S. van Munster, ex *Ruschia pungens*; 1♀, Karoo Desert National Botanical Garden, Worcester, 27.iv.19, N. Dorchin, S. van Munster and C. Klak, ex *Ruschia caroli*; 2♀, 2♂, Karoo Desert National Botanical Garden, Worcester, 14.viii.19, N. Dorchin and S. van Munster, ex *Ruschia caroli*; 4♀, 2♂, Karoo Desert National Botanical Garden, Worcester, 14.viii.19, N. Dorchin and S. van Munster, ex *Lampranthus haworthii*; 1♀, Vrolijkheid Nature Reserve (33°55′04″ S, 19°52′39″ E), 15.viii.19, N. Dorchin, S. van Munster and C. Klak, ex *Ruschia caroli*.

**Other material examined:** 1♂, same data as holotype; 2♀, 1♂, 3 exuviae, Karoo Desert National Botanical Garden, Worcester, 25.viii.17, N. Dorchin, S. van Munster and C. Klak, ex *Ruschia caroli*; 4♀, 3♂, 4 exuviae, Eilandia, Robertson, 15 km W, Rt60, 20.ix.17, N. Dorchin and S. van Munster, ex *Ruschia pungens*; 2 larvae, 5 exuviae, Eilandia, Robertson, 15 km W, Rt60, 4.ix.18, N. Dorchin and S. van Munster, ex *Ruschia caroli*; 8 larvae, 2 exuviae, Eilandia, Robertson, 15 km W, Rt60, 6.ix.18, N. Dorchin and S. van Munster, ex *Ruschia pungens*; 4 larvae, Karoo Desert National Botanical Garden, Worcester, 27.iv.19, N. Dorchin, S. van Munster and C. Klak, ex *Ruschia caroli*; 2♀, 1 exuviae, Karoo Desert National Botanical Garden, Worcester, 14.viii.19, N. Dorchin and S. van Munster, ex *Ruschia caroli*.

### 3.4. Ruschiola attenuata Dorchin and van Munster, New Species

Characters similar to *R. succulenta*, except for the following:

**Hostplant:***Mesembryanthemum splendens* L.

**Gall and biology**: This species induces common inflated, succulent leaf galls, typically up to 2 cm long and 0.5 cm wide (Figure 6E,F). The gall usually occupies the entire leaf, is green, yellowish or reddish, and contains 1–3 chambers located centrally along the longitudinal leaf axis. The walls of the chambers are drier than the surrounding leaf tissue and the gall is firm to the touch. Galls are common throughout the distribution of the host plant and adult gall midges were reared from January to September, suggesting that the species completes multiple generations a year.

**Figure 8:**
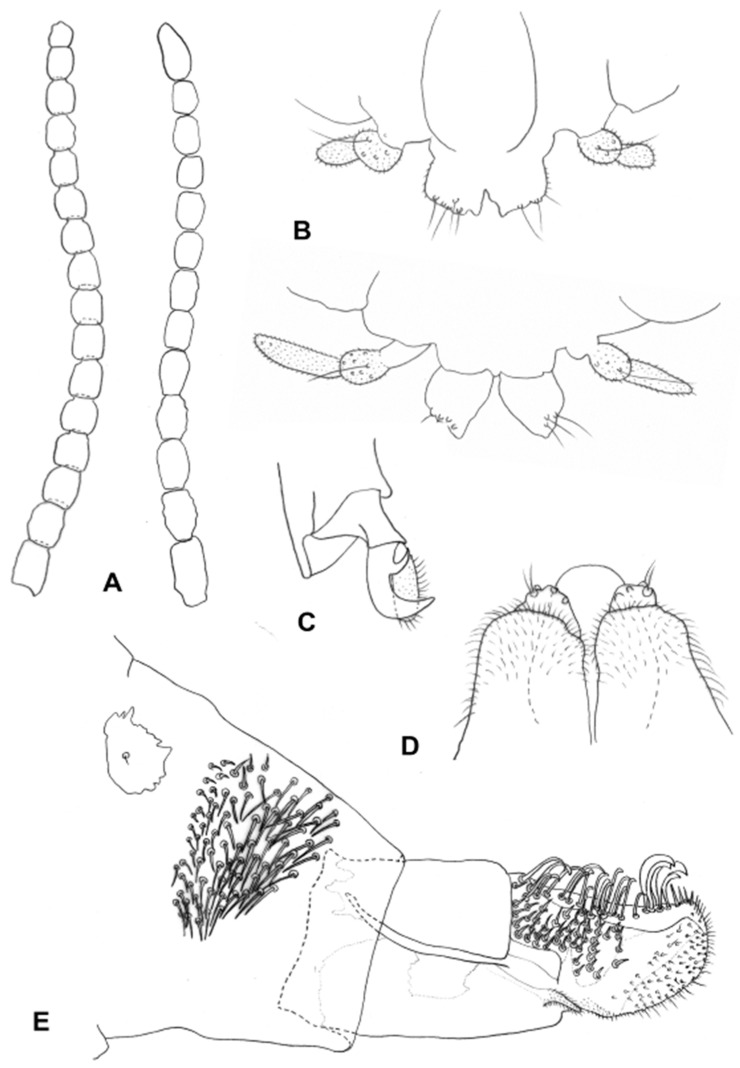
*Ruschiola* spp. (**A**) Antennal flagellomeres; (**right**): *R. succulenta*, (**left**): *R. attenuata*. (**B**) *R. attenuata*, variation in palpus morphology. (**C**) *R. attenuata*, acropod, lateral. (**D**) *R. attenuata*, male aedeagus and mediobasal lobes of gonocoxites, dorsal. (**E**) *R. attenuata*, ovipositor, lateral.

**Adult**: *Head*: Antennal flagellomeres 15–18 in female (*n* = 23), 13–16 in male (*n* = 8), quadrate, about as long as wide, except for slightly longer flagellomeres 1 and 2 (Figure 8A). Palpus two segmented; segment 1 globular, segment 2 as long as or about twice as long as segment 1, when longer usually tapered apically (Figure 8B).

*Thorax*: Wing length: 1.46–2.44 mm in female (*n* = 36), 1.74–2.47 mm in male (*n* = 15). Empodia as long as or slightly longer than bend in claws (Figure 8C).

*Female abdomen*: Tergite 8 with small patch of pigmented area on each side, with anterior sensory seta and sometimes 2–3 small posterior setae. Ovipositor (Figure 8E): Segment 9 with rectangular patch of pigmented area at mid-length. Heavily sclerotized area of lateral plate ventral to curl-like setae thin, sheathing at most quarter height of apical lamella.

*Male abdomen*: Mediobasal lobe of gonocoxite slightly shorter than aedeagus, with shorter dorsal bulge over longer, rugose apical section (Figure 8D).

**Larva**: Not studied.

**Figure 9:**
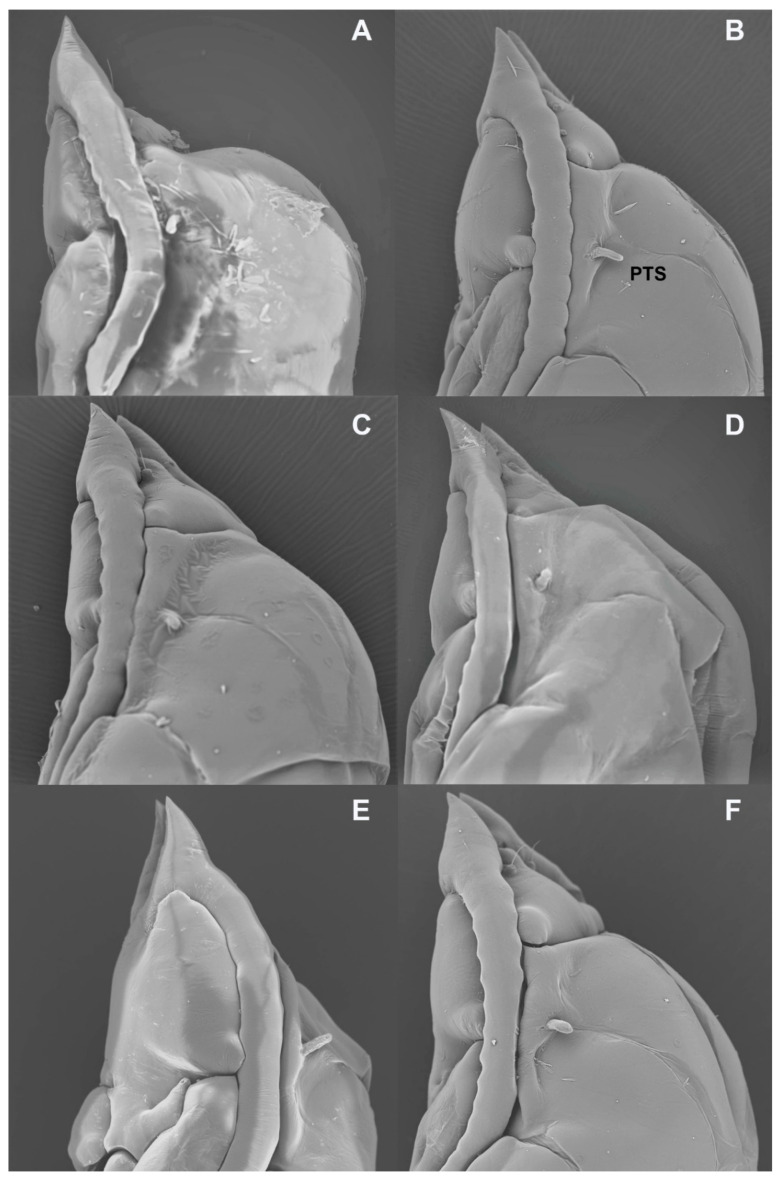
*Ruschiola* pupal heads, lateral. (**A**) *R. attenuata*. (**B**) *R. cedarbergensis*. (**C**) *R. namaqua*. (**D**) *R. bubonis*. (**E**) *R. quagga*. (**F**) *R. leipoldtiae*. PTS: prothoracic spiracle.

**Pupa** (Figure 7B and Figure 9A): Antennal horns straight, separated by triangular gap, each split apically into longer lateral tip and shorter median tip. Face with two tiny pits in mid posterior area.

**Distribution:** Common wherever the host plant was found, outside or along the edge of winter rainfall areas of the Western Cape. Collected from Worcester, Laingsburg, Van Wyksdorp, and Oudtshoorn.

**Etymology:** The species epithet is the Latin feminine adjective term for plain, shortened or refined, with reference to the thinner and less robust lateral plate of the ovipositor compared to the ovipositor of other known *Ruschiola* species.

**Type material:** HOLOTYPE: ♀, South Africa, Western Cape, Van Wyksdorp (Watermill Farm) (33°43′50″ S, 21°28′39″ E), 26.iv.19, N. Dorchin, S. van Munster and C. Klak, ex leaf gall on *Mesembryanthemum splendens*. On permanent microscope slide in Euparal. Deposited in SAMC. PARATYPES: 5♀, 1♂, same data as holotype; 6♀, 1♂, Karoo Desert National Botanical Garden, Worcester, 14.viii.19, N. Dorchin, S. van Munster and C. Klak (1♀ EMEUC); 7♀, 7♂, Laingsburg, 34 km SE, R323 (33°22′52.38″ S, 21°0′41.11″ E), 5.ix.18, J. F. Colville and A. Melin (1♀, 1♂, ZFMK).

**Other material examined:** 4♀, same data as holotype; 1♂, Karoo Desert National Botanical Garden, Worcester (33°36′33″ S, 19°27′01″ E), 14.viii.19, N. Dorchin, S. van Munster and C. Klak; 2♀, 1♂, Oudtshoorn, 27 km S, N12 (33°46′29″ S, 22°20′35″ E), 12.x.18, S. van Munster; 9♀, 4♂, Laingsburg, 34 km SE, R323 (33°22′52.38″ S, 21°0′41.11″ E), 5.ix.18, J. F. Colville and A. Melin.

**Comments:** This is the only *Ruschiola* species that can be readily distinguished from the otherwise rather uniform species in the genus based on its adult morphology. Its flagellomeres are more numerous and significantly shorter than those of the other species described here, the empodia are about as long as the bend in the claws rather than much longer, as in other species, and the ovipositor has a thinner lateral plate and less robust apical lamella. The pupa also stands out among all other species for having apically divided antennal horns, whereas those of all other species are undivided.

### 3.5. Ruschiola cedarbergensis Dorchin and van Munster, New Species

Characters similar to *R. succulenta*, except for the following:

**Host plants:***Ruschia cymosa* L.Bolus, *R. schollii* (Salm-Dyck) Schwantes, *R.* cf. *caroli*, *R.* cf. *cedarbergensis*.

**Figure 10:**
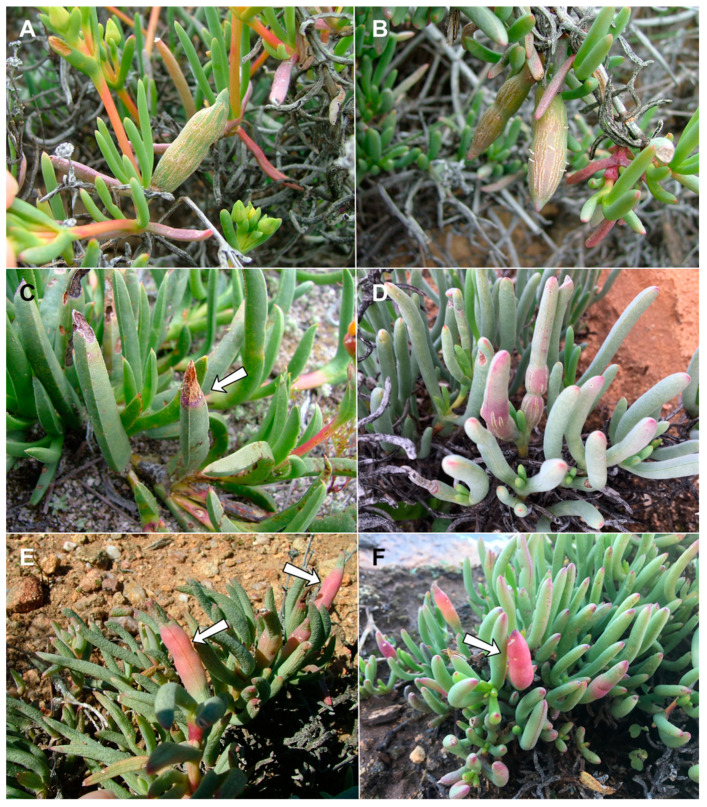
*Ruschiola* galls. (**A**) *R. cedarbergensis* on *Ruschia* cf. *cedarbergensis*. (**B**) *R. cedarbergensis* on *Ruschia cymosa*. (**C**) *R. cedarbergensis* on *R. schollii*. (**D**) *R. cedarbergensis* on *Ruschia* cf. *caroli*. (**E**) *R. namaqua* on *Ruschia goodiae*. (**F**) *R. namaqua* on *R. viridifolia*. Arrows denote galled leaves.

**Gall and biology**: This species induces conspicuous but uncommon sausage-like leaf galls on *R. cymosa*, *R.* cf. *caroli* and *R.* cf. *cedarbergensis* (Figure 10A,B,D), whereas on the low, crawling *R. schollii*, it develops in reddish sections of otherwise undeformed leaves (Figure 10C). The conspicuous galls are usually 5–7 cm long and at least 1 cm wide, often with longitudinal cracks, and may occupy the entire leaf (the common case) or only parts of it. They are firm to the touch and contain many larvae that develop in vaguely defined larval chambers embedded in rigid tissues. Galls were collected in August and September only; it is therefore unknown if this species has more than one generation per year.

**Figure 11:**
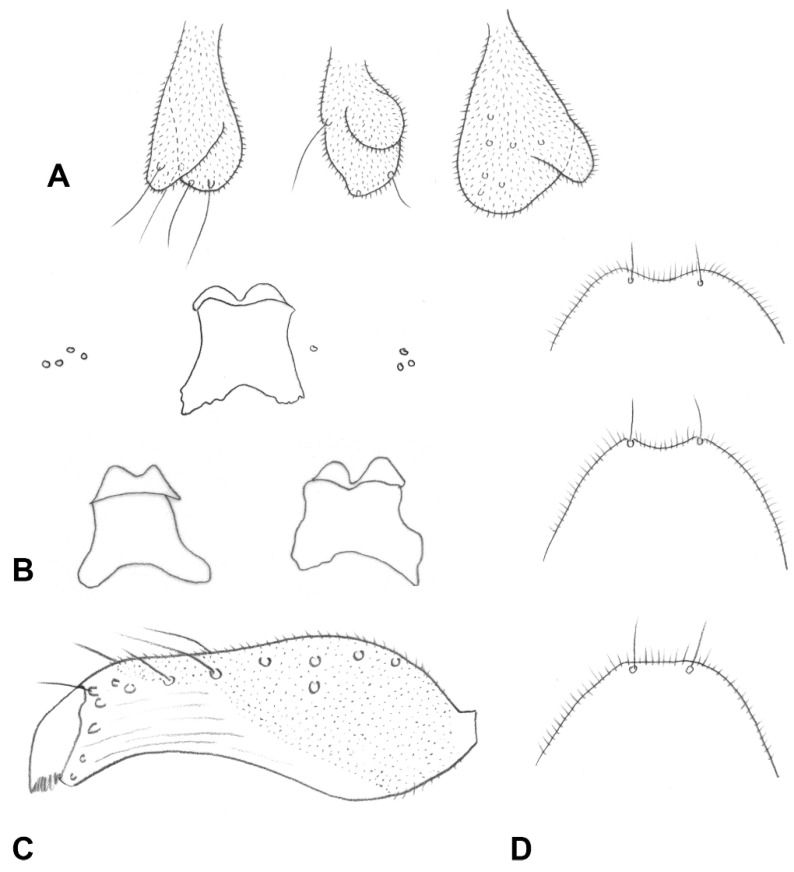
*Ruschiola cedarbergensis*. (**A**) Variation in palpus morphology. (**B**) Variation in spatula shape and associated papillae. (**C**) Male gonostylus, dorsal. (**D**) Variation in shape of male hypoproct.

**Adult**: *Head*: Antennal flagellomeres 12–14 in female (*n* = 24), 11–12 in male (*n* = 10); occasionally two or more flagellomeres fused to form continuous units. Palpus often appears composed of one large, globular segment, but usually with vestigial second segment “riding” on or branching from it (Figure 11A).

*Thorax*: Wing length: 1.99–2.67 mm in female (*n* = 27), 1.99–2.63 mm in male (*n* = 16).

*Female abdomen*: Tergite 8 with small, elongate patch of pigmented area and anterior sensory seta outside of pigmented area.

*Male abdomen*: Gonostylus (Figure 8C) widest at base, with clear constriction around mid-length, distal margin almost straight rather than arched, setulose along basal and most of distal sections, furrowed elsewhere.

**Larva** (third instar): Antennae about twice as long as wide. Spatula (Figure 11B) only slightly longer than wide, shallow teeth rounded apically, short and wide shaft extending into two lobes posteriorly. Lateral papillae asetose. One larva found with long-shafted spatula similar to that of *R. succulenta*; more sampling is needed in order to clarify if the short-shafted larvae represent early third instars with spatula that is not yet fully developed.

**Pupa** (Figure 7C and Figure 9B): Antennal horns separated by triangular gap (median edges not parallel).

**Distribution:** Uncommon on several *Ruschia* species restricted to the Cedarberg region. Recorded from Heuningvlei, Bushmans Kloof Wilderness Reserve, and Travellers Rest (Wolfdrif) near the town of Clanwilliam.

**Etymology:** The species epithet is feminine, combining the name Cedarberg with the Latin adjectival suffix “*ensis*”, which means “from the Cedarberg”, as it was only reared from several host plants in that particular region.

**Type material:** HOLOTYPE: ♂, South Africa, Western Cape, Bushmans Kloof Wilderness Reserve (32°06′22″ S, 19°06′42″ E), 8.viii.19, N. Dorchin and S. van Munster, ex leaf gall on *Ruschia cymosa*. On permanent microscope slide in Euparal. Deposited in SAMC. PARATYPES: 3♀, same data as holotype; 1♀, 1♂, Bushmans Kloof Wilderness Reserve, 14.ix.17, N. Dorchin and S. van Munster, ex. *Ruschia* cf. *caroli*; 3♀, 3♂, Travelers Rest (Wolfdrif), Cedarberg (32°01′47″ S 19°03′19″ E), 11.ix.18, N. Dorchin and S. van Munster, ex. *Ruschia cymosa*; 3♀, 3♂, Bushmans Kloof Wilderness Reserve, 12.ix.18, N. Dorchin and S. van Munster, ex *Ruschia* cf. *cedarbergensis*; 1♀, 1♂, Bushmans Kloof Wilderness Reserve, 12.ix.18, N. Dorchin and S. van Munster, ex. *Ruschia cymosa*; 5♀, Heuningvlei Nature Reserve (32°09′59″ S, 19°01′46″ E), 13.ix.18, N. Dorchin and S. van Munster, ex *Ruschia schollii*; 7♀, 3♂, Bushmans Kloof Wilderness Reserve, 8.viii.19, N. Dorchin and S. van Munster, ex *Ruschia* cf. *caroli*; 3♀, 1♂, Bushmans Kloof Wilderness Reserve, 8.viii.19, N. Dorchin and S. van Munster, ex *Ruschia cymosa*; 4♀, 4♂, Travelers Rest (Wolfdrif), Cedarberg, 8.viii.19, N. Dorchin and S. van Munster, ex *Ruschia* cf. *caroli*.

**Other material examined:** 1 exuviae, same data as holotype; 2 exuviae, Travelers Rest (Wolfdrif), Cedarberg, 11.ix.18, N. Dorchin and S. van Munster, ex. *Ruschia cymosa*; 6 larvae, Bushmans Kloof Wilderness Reserve, 12.ix.18, N. Dorchin and S. van Munster, ex *Ruschia* cf. *cedarbergensis*; 3 larvae, 3 exuviae, Bushmans Kloof Wilderness Reserve, 12.ix.18, N. Dorchin and S. van Munster, ex. *Ruschia cymosa*; 5 exuviae, Bushmans Kloof Wilderness Reserve, 8.viii.19, N. Dorchin and S. van Munster, ex *Ruschia* cf. *caroli*; 4 exuviae, Travelers Rest (Wolfdrif), Cedarberg, 8.viii.19, N. Dorchin and S. van Munster, ex *Ruschia* cf. *caroli*.

**Comments:** The large, succulent leaf galls of this species on most of its host plants are very similar to those caused by *R. succulenta* and the two species appear to be closely related based on molecular data (Figure 2). Nevertheless, slight diagnostic morphological differences do exist between them, including the shorter and more robust palpus in *R. cedarbergensis*, the typical shape of the gonostylus (Figure 11C), the much shorter and wider larval spatula and the more widely separated pupal antennal horns (parallel in *R. succulenta*). It is noteworthy that *R. cedarbergensis* causes large, conspicuous galls on three of its host plants, whereas on *Ruschia schollii*, no obvious galls develop, and the presence of the gall midge in the plant is evident only because of pupal skins that remain stuck in reddish sections of the otherwise undeformed leaves (Figure 10C).

### 3.6. Ruschiola namaqua Dorchin and van Munster, New Species

Characters similar to *R. succulenta*, except for the following:

**Host plants:***Ruschia viridifolia* L.Bolus, and *R. goodiae* L.Bolus.

**Gall and biology**: This species develops in smooth, succulent, reddish leaf galls that usually occupy most of the leaf area (Figure 10E,F). The galls are 3–4 cm long and 1.0–1.5 cm wide, each containing numerous larval chambers, and can be locally very common. They were sampled at several sites in late July to mid-August, at which time they contained second and third instar larvae, pupae, and empty pupal skins. It is currently unknown if this species has more than one generation a year.

**Figure 12:**
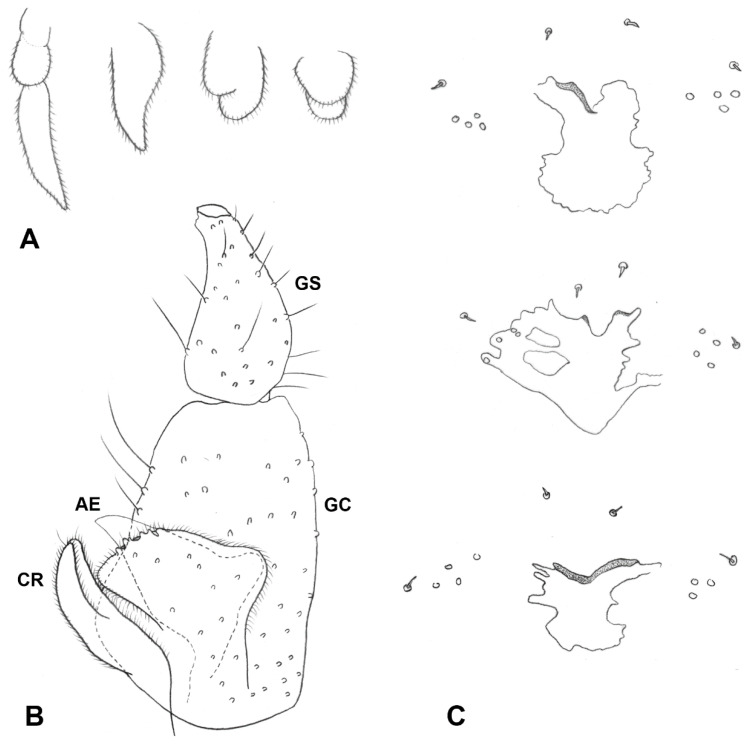
*Ruschiola namaqua*. (**A**) Variation in palpus morphology. (**B**) Male terminalia, lateral. (**C**) Variation in shape of spatula and associated papillae. AE: aedeagus; CR: cerci; GC: gonocoxite; GS: gonostylus.

**Adult**: *Head*: Antennal flagellomeres 12–13 in female (*n* = 39), 10–12 in male (*n* = 35). Palpus usually one segmented, either globular or with tapered extension, sometimes with basal bulge, occasionally two segmented, with tapered second segment much longer than first (Figure 12A).

*Thorax*: Wing length: 1.70–2.39 mm in female (*n* = 39), 1.51–2.20 mm in male (*n* = 36).

*Male abdomen*: Gonostylus evenly setulose along ventral part.

**Larva** (third instar): Spatula (Figure 12C): Vestigial, irregularly pigmented, about as long as wide, without clear teeth or shaft, somewhat more sclerotized anteriorly. Sternal papillae setose, lateral papillae asetose. Pigmented area of spatula sometimes encroaching lateral papillae area.

**Pupa** (Figure 7D and Figure 9C): As described under *S. succulenta*.

**Distribution:** Galls are common on at least two *Ruschia* species in Namaqualand. Collected from Kamieskroon, Grootvlei Pass and Namaqua National Park (Skilpad Rest Camp).

**Etymology:** This species is named after the Namaqualand region, to which it is apparently restricted. The name is a feminine noun in apposition.

**Type material:** HOLOTYPE: ♀, South Africa, Northern Cape, Namaqua National Park (Skilpad Camp), (30°09′58″ S, 17°46′09″ E), 26.vii.19, N. Dorchin, S. van Munster and C. Klak, ex leaf gall on *Ruschia goodiae*. On permanent microscope slide in Euparal, deposited in SAMC. PARATYPES: 9♀, 9♂, same data as holotype. 1♀, 1♂, Kamieskroon (30°12′00″ S, 17°56′06″ E), 9.viii.17, N. Dorchin, S. van Munster and C. Klak, ex *Ruschia viridifolia*; 10♀, 10♂, Grootvlei Pass, eastern base (30°12′53″ S, 17°46′07″ E), 10.viii.17, N. Dorchin, S. van Munster and C. Klak ex *Ruschia goodiae*; 12♀, 18♂, Kamieskroon, 26.vii.19, N. Dorchin and S. van Munster, ex *Ruschia viridifolia* (1♀, 1♂ ZMFK, 1♀, 1♂ EMEUC).

**Other material examined:** 1♀, 2 exuviae, Kamieskroon, 9.viii.2017, N. Dorchin, S. van Munster, C. Klak ex *Ruschia viridifolia*; 4♀, 2♂, 2 exuviae, Grootvlei Pass, eastern base, 10.viii.2017, N. Dorchin, S. van Munster and C. Klak ex *Ruschia goodiae*; 1♀, Grootvlei Pass, 10.viii.2017, N. Dorchin, S. van Munster and C. Klak, ex *Ruschia viridifolia*; 1♀, 2 exuviae, Kamieskroon, 26.vii.2019, N. Dorchin and S. van Munster, ex *Ruschia viridifolia*; three larvae, two exuviae, same data as holotype.

**Comments:** The adults and pupae of this species are virtually indistinguishable from those of *R. succulenta*, whereas the larvae have a greatly reduced spatula relative to the well-developed spatula of *R. succulenta* and setose rather than asetose sternal papillae. The galls are smaller, less rigid and without longitudinal cracks, which are typical of *R. succulenta*, *R. cedarbergensis* and other (undescribed) species from other host plants in Namaqualand.

### 3.7. Ruschiola bubonis Dorchin and van Munster, New Species

Characters similar to *R. succulenta*, except for the following:

**Host plant:***Jordaaniella spongiosa* (L.Bolus) H.E.K. Hartmann.

**Figure 13:**
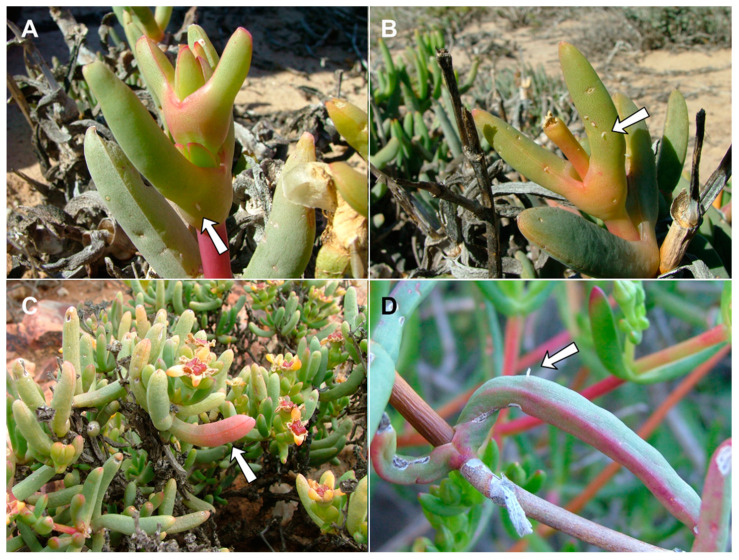
*Ruschiola* galls. (**A**,**B**) *R. bubonis* on *Jordaaniella spongiosa* (arrows: pupal skins in leaves). (**C**) *R. quagga* on *Ruschia holensis*. (arrow: galled leaf) (**D**) *R. timida* on *Scopelogena bruynsii* (arrow: pupal skin in leaf).

**Gall and biology**: This species develops without obvious gall formation in the massive, fleshy leaves of the host plant, and was discovered in the field only due to the empty pupal skins stuck in the leaves (Figure 13A,B). Therefore, plant material was collected haphazardly in an attempt to rear the gall midges, a method that proved successful. Several individuals develop in the same leaf, and the species probably has more than one generation per year, at least during winter, as adults were reared in mid-July and mid-August.

**Figure 14:**
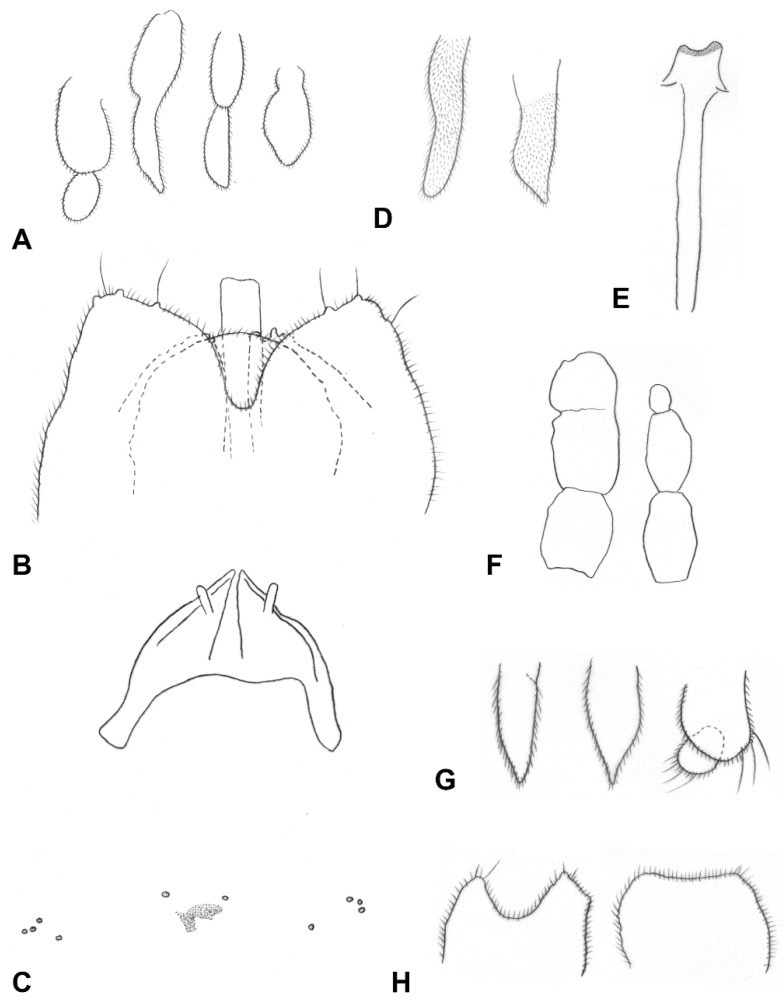
*Ruschiola* spp. (**A**) *R. bubonis*, variation in palpus morphology. (**B**) *R. bubonis*, male cerci, hypoproct, mediobasal lobes and aedeagus, dorsal. (**C**) *R. bubonis*, larval head, spatula and associated papillae. (**D**) *R. quagga*, variation in palpus morphology. (**E**) *R. quagga*, spatula. (**F**) *R. timida*, variation in shape of apical antennal flagellomeres. (**G**) *R. timida*, variation in palpus morphology. (**H**) *R. timida*, variation in shape of male hypoproct.

**Adult**: *Head*: Antennal flagellomeres 12–14 in female (*n* = 10), 11–13 in male (*n* = 5). Palpus morphology variable; occasionally one segmented but usually two segmented, with second segment either shorter or longer than first segment, with long scales (Figure 14A).

*Thorax*: Wing length: 1.24–2.51 mm in female (*n* = 14), 2.00–2.42 mm in male (*n* = 6).

*Female abdomen*: Tergite 8 with small patch of pigmented area or completely undifferentiated from surrounding membrane.

*Male abdomen*: Pigmented area of tergite 8 narrow, band-like or much shorter than preceding. *Terminalia* (Figure 14B): Aedeagus narrow, parallel sided and apically truncated in dorsal view. Hypoproct entire.

**Larva** (third instar) (Figure 14C): Posterolateral apodemes about as long as head capsule. Spatula virtually absent, evident only as small and vaguely defined pigmented area. On each side asetose sternal papilla and four asetose lateral papillae, one of which set closer to median line of body, the other three grouped together. Pleural, dorsal and terminal papillae with short setae.

**Pupa** (Figure 7E and Figure 9D): Antennal horns slightly ventrally arched, widely separated at base, abruptly splayed along median margins from mid-length to tapered apex.

**Distribution:** Locally common on its host plant in the Namaqua National Park (coastal section), the only site where the plant was sampled.

**Etymology:** The epithet *bubonis* is the feminine genitive form of *bubo*—the Latin term for owl—with reference to the splayed antennal horns of the pupa, giving it the appearance of a horned owl (Figure 7E).

**Type material:** HOLOTYPE: ♂, South Africa, Northern Cape, Namaqua National Park (coastal section), (30°24′40″ S, 17°24′59″ E), 28.viii.18, N. Dorchin, S. van Munster and C. Klak, ex *Jordaaniella spongiosa*. On permanent microscope slide in Euparal. Deposited in SAMC. PARATYPES: 4♀, 2♂, same data as holotype; 10♀, 3♂, Namaqua National Park (coastal section), 25.vii.19, N. Dorchin, S. van Munster and C. Klak.

**Other material examined:** 1 larva, 3 exuviae, same data as holotype.

**Comments:** This species stands out among congeners with known pupae for the splayed, owl-like antennal horns of its pupa compared to the triangular or parallel-sided antennal horns of other species. Females cannot be distinguished from those of other species, with the exception of *R. attenuata*, but males have a consistently entire hypoproct, whereas the shape of the hypoproct in other species varies. The single available larva lacks a distinct spatula, and its pleural, dorsal and terminal papillae bear much shorter setae than those of other species.

### 3.8. Ruschiola quagga Dorchin and van Munster, New Species

Characters similar to *R. succulenta*, except for the following:

**Host plant:***Ruschia holensis* L. Bolus.

**Gall and biology**: This species develops in inflated leaf galls, 2–3 cm long and about 1 cm wide, green to reddish (Figure 13C). Each gall contains a few larvae. In young galls containing second instar larvae, the larvae are found in vaguely differentiated chambers embedded in the spongy leaf tissue. Third instar larvae and pupae are found in more defined chambers with drier walls compared to the juicy surrounding tissues. The galls are heavily parasitized by polyembryonic hymenopteran parasitoids. Galls were collected only once, in late August, at which time they contained either second or third instar larvae and pupae. It is unknown if the species has more than one generation per year.

**Adult**: *Head*: Antennal flagellomeres 13 in female (*n* = 3), unknown in male (no males with complete antennae). Palpus one segmented, 1.3–5.5 times as long as wide, usually tapered (Figure 14D); when short, tear shaped and based on small palpiger; shape may differ between palpi of same individual.

*Thorax*: Wing length: 2.2–2.33 mm in female (*n* = 8), 1.81–1.97 mm in male (*n* = 3).

*Male abdomen*: Hypoproct truncated apically.

**Larva** (third instar): Antennae about 1.5 times as long as wide. Posterolateral apodemes about as long as head capsule. Spatula with shallow depression between short and blunt teeth, long and narrow shaft (Figure 13E). Sternal and lateral papillae asetose, barely visible. Pleural, dorsal and terminal papillae with minute setae.

**Pupa** (Figure 9E): As described under *R. succulenta*.

**Distribution:** Found only once in the Knersvlakte (Quaggaskop Farm).

**Etymology:** This species is named after the extinct South African Plains Zebra—Quagga. It was collected only at Quaggaskop Farm in the Knersvlakte. The name is a feminine noun in apposition.

**Type material:** HOLOTYPE: ♀, South Africa, Western Cape, Quaggaskop Farm, Knersvlakte Nature Reserve (31°24′59″ S, 18°35′43″ E), 26.viii.18, N. Dorchin, S. van Munster and C. Klak, ex leaf gall on *Ruschia holensis*. On permanent microscope slide in Euparal. Deposited in SAMC. PARATYPES: 7♀, 3♂, 2 larvae, 2 exuviae, same data as holotype.

### 3.9. Ruschiola timida Dorchin and van Munster, New Species

Characters similar to *R. succulenta*, except for the following:

**Host plant:***Scopelogena bruynsii* Klak.

**Gall and biology**: This species is rare, or at least very difficult to find. It develops without causing any obvious deformation in the leaves of its host plant and was reared only by collecting plant material haphazardly after finding a small number of empty pupal skins stuck in leaves (Figure 13D). On one occasion, a slightly inflated leaf was found, but all adults that we reared emerged from leaves with no external signs of infestation. Adults were reared only from one site, although we sampled the host plant at other sites within the same area, where the plant is rather common. In 2017 and 2018, plants were collected and adults emerged from them in mid-September; in 2019 plants were sampled in early August, but no adults emerged. Additional sampling of the host plant is needed at different times of the year to confirm if the species has only one generation in early spring.

**Adult**: *Head*: Antennal flagellomeres 13–14 in female (*n* = 9), 10–12 in male (*n* = 3); apical flagellomere often “budding” from penultimate flagellomere or two apical flagellomeres fused (Figure 14F). Palpus usually one segmented, tapered, about twice as long as wide, or two segmented, with second segment much smaller than first (Figure 14G).

*Thorax*: Wing length: 1.95–2.42 mm in female (*n* = 9), 1.68–1.93 mm in male (*n* = 4).

*Male abdomen*: Hypoproct truncated or with deep notch (Figure 14H).

**Larva**: Unknown.

**Pupa**: Unknown.

**Distribution:** This species was found on only a few occasions in the Cedarberg (Travellers Rest).

**Etymology:** The species name is a feminine adjective derived from the Latin term for timid, referring to the lack of external signs of its presence in the leaves of the host plant and the difficulty of finding and rearing it.

**Type material:** HOLOTYPE: ♀, South Africa, Western Cape, Travellers Rest, Clanwiliam (32°05′03″ S, 19°05′24″ E), 13.ix.18, N. Dorchin and S. van Munster, ex leaf of *Scopelogena bruynsii*. On permanent microscope slide in Euparal. Deposited in SAMC. PARATYPES: 6♀, 4♂ same data as holotype. 2♀ Travellers Rest, Clanwilliam, 15.ix.17, N. Dorchin and S. van Munster.

### 3.10. Ruschiola furtiva Dorchin and van Munster, New Species

Characters similar to *R. succulenta*, except for the following:

**Host plant:***Ruschia dichroa* (Rolfe) L. Bolus.

**Gall and biology**: This species develops in inconspicuous leaf galls, the only external deformation of the leaf being a slight change of color from green to red. The presence of the gall midges in the leaves was recognized only by the empty pupal skins that were found stuck in slightly discolored leaves. Although the same site was visited twice in subsequent years, and host plant material was collected, gall midges were reared only once, in mid-September; no other information is currently available on the life history of this species.

**Adult**: *Head*: Antennal flagellomeres 12–13 in female (*n* = 4), 11–12 in male (*n* = 4). Palpus one segmented, 2–4 times as long as wide, tapered apically.

*Thorax*: Wing length: 2.13–2.36 mm in female (*n* = 5), 1.83–1.93 mm in male (*n* = 4).

*Female abdomen*: Tergite 8 undifferentiated from surrounding tissue.

*Male abdomen*: Gonostylus completely setulose dorsally and ventrally. Hypoproct truncated.

**Larva**: Unknown.

**Pupa**: Similar to that of *R. succulenta*, based on pupal exuviae.

**Distribution:** This species was found only once in Bushmans Kloof Wilderness Reserve in the Cedarberg region, to which it is probably endemic, similar to its host plant [40].

**Etymology:** The species epithet is a feminine Latin adjective for secret or hidden, referring to the lack of external signs of its presence in the leaves of its host plant.

**Type material:** HOLOTYPE: ♀, South Africa, Western Cape, Bushmans Kloof Wilderness Reserve (32°06′22″ S, 19°06′42″ E), 14.ix.17, N. Dorchin and S. van Munster, ex leaf gall on *Ruschia dichroa*. On permanent microscope slide in Euparal. Deposited in SAMC. PARATYPES: 4♀, 4♂, same data as holotype.

**Other material examined:** 8 exuviae, same data as holotype.

### 3.11. Ruschiola leipoldtiae Dorchin and van Munster, New Species

Characters similar to *R. succulenta*, except for the following:

**Host plant:***Leipoldtia laxa* L. Bolus and *L. schultzei* (Schltr. and Diels) Friedrich.

**Figure 15:**
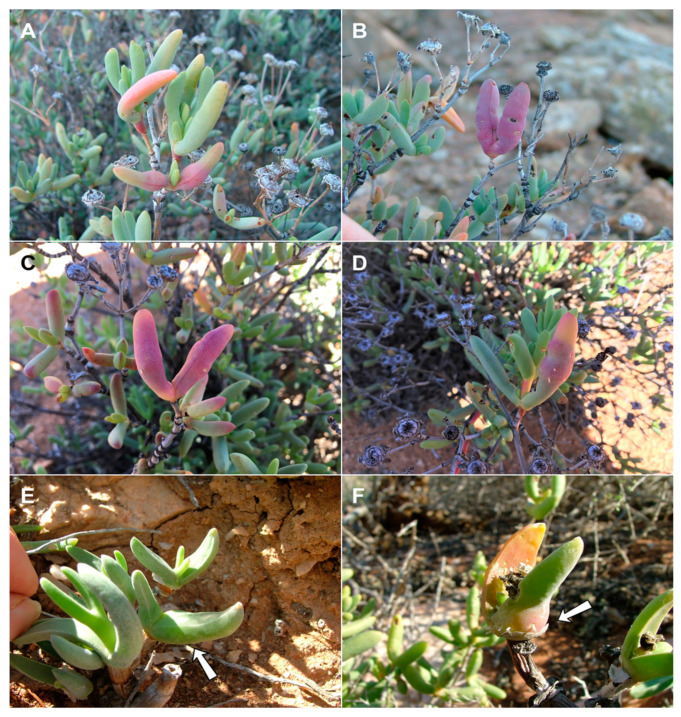
*Ruschiola* galls. (**A**,**B**) *R. leipoldtiae* on *Leipoldtia laxa*. (**C**,**D**) *R. leipoldtiae* on *Leipoldtia scultzei*. (**E**,**F**) *R. celebrata* on *Mitrophyllum mitratum*. Arrows: galled part of leaf.

**Gall and biology**: This species induces common leaf galls on its host plants, which occupy either the entire leaf or only part of it (Figure 15A–D). The galls are pinkish-red, rigid, and contain multiple larval chambers (10–20 on *L. schultzei*, fewer on *L. laxa*) embedded in a mass of tough tissue in the center of the fleshy leaf. Galls were found and midges were reared in late July and early August. No additional information is available on the life history of this species; however, in December no galls were found on *L. laxa* at the same site from which they were collected in August of the previous year; hence, the species appears to be inactive during the summer.

**Figure 16:**
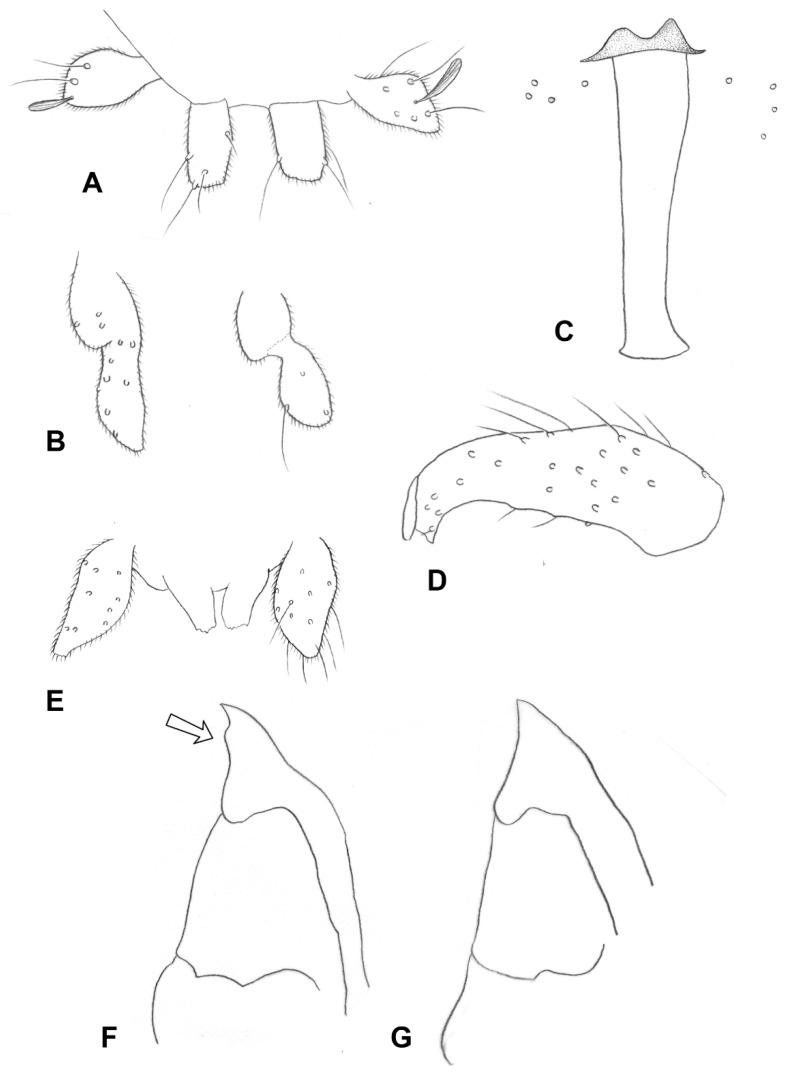
*Ruschiola* spp. (**A**) *R. leipoldtiae*, palpi and labella. (**B**) *R. leipoldtiae*, variation in palpus morphology. (**C**) *R. leipoldtiae*, larval spatula and associated papillae. (**D**) *R. leipoldtiae*, male gonostylus, dorsal. (**E**) *R. celebrata*, palpi and labella. (**F**) *R. celebrata*, pupal exuviae, head, lateral. (Arrow: bulge on antennal horn). (**G**) *R. succulenta*, pupal exuviae, head, lateral.

**Adult**: *Head*: Antennal flagellomeres 11–13 in female (*n* = 26), 11–12 in male (*n* = 10). Palpus one or two segmented (Figure 16A,B), number sometimes varies in same individual; when one segmented, segment ovoid, 1.5–2.0 times as long as wide; when two segmented, segment 2 subequal to or longer than segment 1 and appears to “ride” on it (Figure 16B). Labella prominent and elongated, 1.5 times as long as wide (Figure 16A).

*Thorax*: Wing length: 1.93–2.32 mm in female (*n* = 44), 1.60–2.18 mm in male (*n* = 38).

*Male abdomen*: Gonostylus widest at proximal third, distal margin almost straight rather than arched (Figure 16D). Hypoproct truncated or with shallow notch.

**Larva** (third instar): Posterolateral apodeme about as long as head capsule. Spatula well developed, long shafted (Figure 16C). On each side of spatula asetose sternal papilla and 4 asetose lateral papillae, one of which set further apart from the other three.

**Pupa** (Figure 10F and Figure 11F): Antennal horns separated by triangular gap (median edges not parallel).

**Distribution:** This species was found in Springbok and in the Namaqua National Park (Skilpad camp), suggesting that it has a wide distribution in Namaqualand.

**Etymology:** This species is named after its host plant genus *Leipoldtia*, which in turn is named after the renowned South African physician, poet and botanist, C. Louis Leipoldt (1880–1947). The epithet is a feminine noun in the genitive case.

**Type material:** HOLOTYPE: ♂, South Africa, Northern Cape, Namaqua National Park (Skilpad camp) (30°09′58″ S, 17°46′09″ E), 21.vii.19, N. Dorchin, S. van Munster and C. Klak, ex leaf gall on *Leipoldtia schultzei*. On permanent microscope slide in Euparal. Deposited in SAMC. PARATYPES: 12♀, 12♂, same data as holotype; 17♀, 10♂, Springbok (29°40′53″ S 17°53′03″ E), 5.viii.17, N. Dorchin, S. van Munster and C. Klak.

**Other material examined:** 4♀, 15♂, same data as holotype; 14♀, 7 larvae, 10 exuviae, Springbok, 5.viii.2017, N. Dorchin, S. van Munster and C. Klak.

**Comments:** This species stands out among its congeners for its conspicuous labella and dense cover of hair-like setae on the adult frons and male gonopods. The gonostylus is straight along its posterior margin rather than arched, as in most species. The pupa resembles that of *R. cedarbergensis* in having antennal horns that are not parallel along their median margins (although this character depends on the angle from which the pupae are viewed and may be distorted in slide-mounted exuviae).

### 3.12. Ruschiola celebrata Dorchin and van Munster, New Species

Characters similar to *R. succulenta*, except for the following:

**Host plants:***Mitrophyllum mitratum* (Marloth) Schwantes, *M. clivorum* (N.E.Br.) Schwantes.

**Gall and biology**: This species was reared from inflated leaf galls that usually occupy the base of the leaf, but which occasionally occupy its entire length (Figure 15E,F). Often only one of the two leaves on the same node is affected (Figure 15E). The galls were locally very common on the two host plants at a particular spot, but absent from other spots at the same site. As would be expected, galls were larger on *M. mitratum* (2–4 cm) than on the smaller and more delicate *M. clivorum* (1–2 cm). Each gall contained 10–30 larvae, as attested by the large numbers of empty pupal skins that were stuck in them. The galls are not particularly harder than the soft, uninfested leaves, and most are green but sometimes yellowish. This species was observed and reared only once, in late July, and no additional information is available on its life history.

**Adult**: *Head*: Antennal flagellomeres 12–15 in female (*n* = 13), 11–12 in male (*n* = 2). Palpus one segmented, 1.5–4 times as long as wide, fusiform with truncated apex (Figure 16E).

*Thorax*: Wing length: 2.22–2.73 mm in female (*n* = 13), 2.13–2.44 mm in male (*n* = 3).

*Female abdomen*: Tergite 8 with small patch of heavily pigmented area.

**Larva**: Unknown.

**Pupa** (based on exuviae): Antennal horns with small subapical bulge (Figure 16F).

**Distribution:** This species was found on only one occasion at the south-eastern end of the Vyftienmyl se Berg Inselberg, ca. 30 km east of Port Nolloth.

**Etymology: The** species epithet is a feminine Latin adjective for crowded or populous, with reference to the large number of larvae in the gall.

**Type material:** HOLOTYPE: ♀, South Africa, Northern Cape, Vyftienmyl se Berg Inselberg, Port Nolloth, 20 km E (29°14′41″ S, 17°06′32″ E), 22.vii.19, N. Dorchin, S. van Munster and C. Klak, ex leaf gall on *Mitrophyllum* *clivorum*. On permanent microscope slide in Euparal. Deposited in SAMC. PARATYPE: 5♀, 3♂, same data as holotype. 7♀, same data as holotype, from *Mitrophyllum mitratum*.

**Other material examined:** 4 exuviae, same data as holotype; 3 exuviae, same data as holotype, from *Mitrophyllum mitratum*.

**Comments:** The pupa of this species is unique among other known pupae in this genus for having a small but distinct bulge just below the apex of the antennal horn (compare Figure 16F,G).

## 4. Discussion and Conclusions

In this study we revealed a species-rich community of gall midges on southern African Aizoaceae, which was unknown to date. While it was obvious that these gall midges belong to the Lasiopterini, they were morphologically distinct from all other known genera in that tribe, justifying the description of a new genus. Our findings attest to the vast gap in the knowledge on Afrotropical Cecidomyiidae and on the diversity of Lasiopterini in particular, the greatest diversity of which is currently known from Chenopodiaceae host plants in the Palaearctic region [9,21,41]. The phylogenetic relations of *Ruschiola* to other genera in the Lasiopterini require further study.

The general morphological uniformity among *Ruschiola* species may indicate a recent diversification on succulent Aizoaceae, independently from the parallel diversification of *Asphondylia* species on these host plants. This parallel diversification was probably aided by the fact that the two taxa occupy different niches on the host plants, with the *Ruschiola* species developing in leaves and the *Asphondylia* species in buds. Most of the *Ruschiola* species described here are highly host specific, having been recorded from one or a few closely related host-plant species, as seen in Lasiopterini from Chenopodiaceae (e.g., [38,42]). However, the two groups differ in that chenopod-associated taxa are much more diverse morphologically and that each chenopod host often supports multiple rather than a single lasiopterine species (e.g., [43,44,45]), including in South Africa (N. Dorchin, unpublished data). These observations suggest that the association of Lasiopterini with Chenopodiaceae is older than their association with Aizoaceae.

The phylogenetic inference presented here implies that the cecidomyiid species associated with *Ruschia* spp. are more closely related to each other than to species from other host plant taxa, although these results should be taken with caution given that they are based on a single mitochondrial gene. They also attest to a strong geographic effect, by which the same cecidomyiid species uses several host plant taxa that are found in physical proximity (e.g., *R. cedarbergensis* and *R. namaqua* on several *Ruschia* species in the Cedarberg and Namaqualand regions, respectively). Lastly, our findings provide yet another example of the importance of immature stages in studies of taxonomically difficult cecidomyiid groups, as larvae and pupae may provide diagnostic characters that are absent in adults (e.g., [46,47,48]).

## Figures and Tables

**Table 1:** Individuals used in the molecular analysis with the data collection information and GenBank accession numbers. All individuals were collected in South Africa (WC: Western Cape; NC: Northern Cape).

Sample Name	Host Plant (Voucher No.)	Locality	GenBank Accession Number
*Ruschiola attenuata* 152	*Mesembryanthemum splendens* (Colville and Melin 152, BOL)	Laingsburg, 34 km S, R323, WC, 33.37337 S 21.11395 E	OL415485
*Ruschiola attenuata* 152-2	*Mesembryanthemum splendens* (Colville and Melin 152, BOL)	Laingsburg, 34 km S, R323, WC, 33.37337 S 21.11395 E	OL415484
*Ruschiola attenuata* 167	*Mesembryanthemum splendens* (S. van Munster 167, BOL)	Oudtshoorn, 27 km S, N12, WC, 33.77472 S 20.34306 E	OL415482
*Ruschiola attenuata* 167-2	*Mesembryanthemum splendens* (S. van Munster 167, BOL)	Oudtshoorn, 27 km S, N12, WC, 33.77472 S 20.34306 E	OL415481
*Ruschiola attenuata* 210	*Mesembryanthemum splendens* (S. van Munster, N. Dorchin and C. Klak 210, BOL)	Van Wyksdorp (Watermill Farm), Little Karoo, WC, 33.73056 S 21.47750 E	OL415483
*Ruschiola attenuata* 276	*Mesembryanthemum splendens* (S. van Munster, N. Dorchin and J. Colville 99, BOL)	Karoo Desert National Botanical Garden, Worcester, WC, 33.61194 S 19.44972 E	OL415480
*Ruschiola bubonis* 129	*Jordaaniella spongiosa* (Klak 1842, BOL)	Namaqua National Park, Coastal Gate, NC, 30.26290 S 17.25200 E	OL415479
*Ruschiola bubonis* 129-2	*Jordaaniella spongiosa* (Klak 1842, BOL)	Namaqua National Park, Coastal Gate, NC, 30.26290 S 17.25200 E	OL415478
*Ruschiola cedarbergensis* 141	*Ruschia cymosa* (S. van Munster 141, BOL)	Travelers Rest (Wolfdrif), Clanwilliam, WC, 32.02972 S 19.05528 E	OL415458
*Ruschiola cedarbergensis* 147	*Ruschia cf cedarbergensis* (S. van Munster 147, BOL)	Bushmans Kloof, WC, 32.10556 S 19.11083 E	OL415463
*Ruschiola cedarbergensis* 148	*Ruschia cymosa* (S. van Munster 148, BOL)	Bushmans Kloof Wilderness Reserve, WC, 32.12056 S 19.10778 E	OL415456
*Ruschiola cedarbergensis* 149	*Ruschia schollii* (S. van Munster and N. Dorchin 270, BOL)	Heuningvlei Nature Reserve, WC, 32.15833 S 19.03194 E	OL415457
*Ruschiola cedarbergensis* 265-1	*Ruschia cf caroli* (S. van Munster and N. Dorchin 265, BOL)	Bushmans Kloof Wilderness Reserve, WC, 32.12056 S 19.10778 E	OL415454
*Ruschiola cedarbergensis* 90	*Ruschia cf caroli* (S. van Munster, N. Dorchin and A. Magee 90, BOL)	Bushmans Kloof Wilderness Reserve, WC, 32.08250 S 19.10389 E	OL415461
*Ruschiola cedarbergensis* 265-2	*Ruschia cf caroli* (S. van Munster and N. Dorchin 265, BOL)	Bushmans Kloof Wilderness Reserve, WC, 32.12056 S 19.10778 E	OL415455
*Ruschiola cedarbergensis* 266	*Ruschia cymosa* (S. van Munster and N. Dorchin 266, BOL)	Bushmans Kloof, WC, 32.12056 S 19.10778 E	OL415462
*Ruschiola cedarbergensis* 267-1	*Ruschia cf caroli* (S. van Munster and N. Dorchin 267, BOL)	Travelers Rest (Wolfdrif), Clanwilliam, WC, 32.02972 S 19.05528 E	OL415459
*Ruschiola cedarbergensis* 267-2	*Ruschia cf caroli* (S. van Munster and N. Dorchin 267, BOL)	Travelers Rest (Wolfdrif), Clanwilliam, WC, 32.02972 S 19.05528 E	OL415460
*Ruschiola celebrata* 232-2	*Mitrophyllum mitratum* (S. van Munster, N. Dorchin and C. Klak 232, BOL)	Vyftienmyl se Berg Inselberg, NC, 29.24495 S 17.10896 E	OL415475
*Ruschiola celebrata* 233-1	*Mitrophyllum clivorum* (S. van Munster, N. Dorchin and C. Klak 233, BOL)	Vyftienmyl se Berg Inselberg, NC, 29.24495 S 17.10896 E	OL415477
*Ruschiola celebrata* 233-2	*Mitrophyllum clivorum* (S. van Munster, N. Dorchin and C. Klak 233, BOL)	Vyftienmyl se Berg Inselberg, NC, 29.24495 S 17.10896 E	OL415476
*Ruschiola furtiva* 88-1	*Ruschia dichroa* (S. van Munster, N. Dorchin and C. Klak 88, BOL)	Bushmans Kloof Wilderness Reserve, WC, 32.10639 S 19.11167 E	OL415439
*Ruschiola furtiva* 88-2	*Ruschia dichroa* (S. van Munster, N. Dorchin and C. Klak 88, BOL)	Bushmans Kloof Wilderness Reserve, WC, 32.10639 S 19.11167 E	OL415440
*Ruschiola leipoldtiae* 2-1	*Leipoldtia laxa* (S. van Munster, N. Dorchin and C. Klak 2, BOL)	Springbok, NC, 29.68139 S 17.88417 E	OL415470
*Ruschiola leipoldtiae* 2-2	*Leipoldtia laxa* (S. van Munster, N. Dorchin and C. Klak 2, BOL)	Springbok, NC, 29.68139 S 17.88417 E	OL415471
*Ruschiola leipoldtiae* 245-1	*Leipoldtia schultzei* (S. van Munster, N. Dorchin and C. Klak 245, BOL)	Namaqua National Park, Skilpad Camp, NC, 30.16611 S 17.76917 E	OL415474
*Ruschiola leipoldtiae* 245-2	*Leipoldtia schultzei* (S. van Munster, N. Dorchin and C. Klak 245, BOL)	Namaqua National Park, Skilpad Camp, NC, 30.16611 S 17.76917 E	OL415473
*Ruschiola leipoldtiae* 245-3	*Leipoldtia schultzei* (S. van Munster, N. Dorchin and C. Klak 245, BOL)	Namaqua National Park, Skilpad Camp, NC, 30.16611 S 17.76917 E	OL415472
*Ruschiola namaqua* 30	*Ruschia viridifolia* (S. van Munster, N. Dorchin and C. Klak 30, BOL)	Kamieskroon, NC, 30.19778 S 17.93611 E	OL415446
*Ruschiola namaqua* 31	*Ruschia viridifolia* (S. van Munster, N. Dorchin and C. Klak 31, BOL)	Namaqua National Park, Skilpad, Camp, NC, 30.21472 S 17.76861 E	OL415453
*Ruschiola namaqua* 32-1	*Ruschia goodiae* (S. van Munster, N. Dorchin and C. Klak 32, BOL)	Grootvlei Pass, NC, 30.21611 S 17.75028 E	OL415451
*Ruschiola namaqua* 32-2	*Ruschia goodiae* (S. van Munster, N. Dorchin and C. Klak 32, BOL)	Grootvlei Pass, NC, 30.21611 S 17.75028 E	OL415449
*Ruschiola namaqua* 244-1	*Ruschia viridifolia* (S. van Munster, N. Dorchin and C. Klak 30, BOL)	Kamieskroon, NC, 30.19778 S 17.93611 E	OL415445
*Ruschiola namaqua* 244-2	*Ruschia viridifolia* (S. van Munster, N. Dorchin and C. Klak 30, BOL)	Kamieskroon, NC, 30.19778 S 17.93611 E	OL415448
*Ruschiola namaqua* 246-1	*Ruschia goodiae* (S. van Munster, N. Dorchin and C. Klak 246, BOL)	Namaqua National Park, Skilpad Camp, NC, 30.16611 S 17.76917 E	OL415452
*Ruschiola namaqua* 246-1-20	*Ruschia goodiae* (S. van Munster, N. Dorchin and C. Klak 246, BOL)	Namaqua National Park, Skilpad Camp, NC, 30.16611 S 17.76917 E	OL415447
*Ruschiola namaqua* 246-2-20	*Ruschia goodiae* (S. van Munster, N. Dorchin and C. Klak 246, BOL)	Namaqua National Park, Skilpad Camp, NC	OL415450
*Ruschiola quagga* 121	*Ruschia holensis* (S. van Munster 121, BOL)	Quaggaskop Farm, Knersvlakte, WC, 31.44861 S 18.56833 E	OL415444
*Ruschiola quagga* 121-2	*Ruschia holensis* (S. van Munster 121, BOL)	Quaggaskop Farm, Knersvlakte, WC, 31.44861 S 18.56833 E	OL415443
*Ruschiola succulenta* 50-1	*Ruschia caroli* (S. van Munster, N. Dorchin and Klak 50, BOL)	Karoo Desert National Botanical Garden, Worcester, WC, 33.97000 S 19.65167 E	OL415464
*Ruschiola succulenta* 94-1	*Ruschia caroli* (S. van Munster, N. Dorchin and C. Klak 94, BOL)	Karoo Desert National Botanical Garden, Worcester, WC, 33.61194 S 19.44972 E	OL415469
*Ruschiola succulenta* 94-2	*Ruschia caroli* (S. van Munster, N. Dorchin and C. Klak 94, BOL)	Karoo Desert National Botanical Garden, Worcester, WC, 33.61194 S 19.44972 E	OL415468
*Ruschiola succulenta* 95-2	*Lampranthus haworthii* (S. van Munster, N. Dorchin and C. Klak 95, BOL)	Karoo Desert National Botanical Garden, Worcester, WC, 33.61194 S 19.44972 E	OL415467
*Ruschiola succulenta* 100	*Ruschia caroli* (S. van Munster, N. Dorchin and J. Colville 100, BOL)	Eilandia, near Worcester, WC, 33.77083 S 19.74806 E	OL415466
*Ruschiola succulenta* 101-1	*Ruschia pungens* (S. van Munster, N. Dorchin and J. Colville 101, BOL)	Eilandia, near Worcester, WC, 33.77083 S 19.74806 E	OL415465
*Ruschiola timida* 91	*Scopelogena bruynsii* (S. van Munster, N. Dorchin and C. Klak 91, BOL)	Travelers Rest, Clanwilliam, WC, 32.08416 S 19.09000	OL415442
*Ruschiola timida* 142	*Scopelogena bruynsii* (S. van Munster, N. Dorchin and C. Klak 91, BOL)	Travelers Rest, Clanwilliam, WC, 32.08416 S 19.09000 E	OL415441
**Outgroups**			
*Asteromyia carbonifera*	*Solidago altissima*	USA, MD, Silver Spring	MN191258
*Baldratia salicorniae*	*Sarcocornia perennis*	Israel, Akko	MN191262
*Lasiopera arundinis*	*Arundo donax*	Israel, Hod Hasharon	MN191311
*Lasioptera carophila*	*Foeniculum vulgare*	Israel, Kfar Hahoresh	MN191366
*Lasioptera rubi*	*Rubus fruticosus*	Germany, NRW, Ramersdorf	MN191313
*Ozirhincus longicollis*	*Anthemis bornmuelleri*	Israel, Maagar Bental	MN191363
*Stefaniola* sp. 89	*Haloxylon persicum*	Israel, Yotvata	OL415486
*Suaediola quotidiana*	*Suaeda fruticosa*	Israel, Enot Zuqim	MN191315

**Table 2:** Uncorrected pairwise sequence divergences among and within the ten described *Ruschiola* species, calculated using Paup* [39]. Intraspecific divergences are highlighted in grey.

	*R. succulenta*	*R. attenuata*	*R. cedarbergensis*	*R. namaqua*	*R. bubonis*	*R. quagga*	*R. timida*	*R. furtiva*	*R. leipoldtiae*	*R. celebrata*
*R. succulenta*	0.002									
*R. attenuata*	0.084	0.002								
*R. cedarbergensis*	0.046	0.099	0.007							
*R. namaqua*	0.061	0.083	0.069	0.004						
*R. bubonis*	0.075	0.067	0.102	0.088	0.000					
*R. quagga*	0.058	0.091	0.070	0.026	0.090	0.000				
*R. timida*	0.055	0.081	0.078	0.054	0.081	0.062	0.000			
*R. furtiva*	0.069	0.088	0.087	0.078	0.082	0.082	0.072	0.000		
*R. leipoldtiae*	0.057	0078	0.081	0.060	0.078	0.073	0.051	0.067	0.005	
*R. celebrata*	0.058	0.071	0.074	0.065	0.081	0.069	0.055	0.056	0.052	0.012

## Data Availability

Sequences were deposited in GenBank—respective accession numbers are provided in Table 1. Other data are available upon request from the corresponding author.
